# Sensoproteomic Characterization of *Lactobacillus Johnsonii*-Fermented Pea Protein-Based Beverage: A Promising Strategy for Enhancing Umami and Kokumi Sensations while Mitigating Bitterness

**DOI:** 10.1021/acs.jafc.4c02317

**Published:** 2024-07-03

**Authors:** Andrea Spaccasassi, Lijuan Ye, Cristian Rincón, Rosa Aragao Börner, Biljana Bogicevic, Arne Glabasnia, Thomas Hofmann, Corinna Dawid

**Affiliations:** † Chair of Food Chemistry and Molecular and Sensory Science, TUM School of Life Sciences, Technical University of Munich, Lise-Meitner-Str. 34, Freising 85354, Germany; ‡ TUM CREATE, 1 CREATE Way, #10-02 CREATE Tower, Singapore 138602, Singapore; § Société des Produits Nestlé S.A., Nestlé Research, Route du Jorat 57, Lausanne 26 CH 1000, Switzerland; ⊥ Professorship for Functional Phytometabolomics, TUM School of Life Sciences, Technical University of Munich, Lise-Meitner-Str. 34, Freising 85354, Germany

**Keywords:** Fermentation of pea protein, L. johnsonii, taste improvement, kokumi and umami, senso(prote)omics.

## Abstract

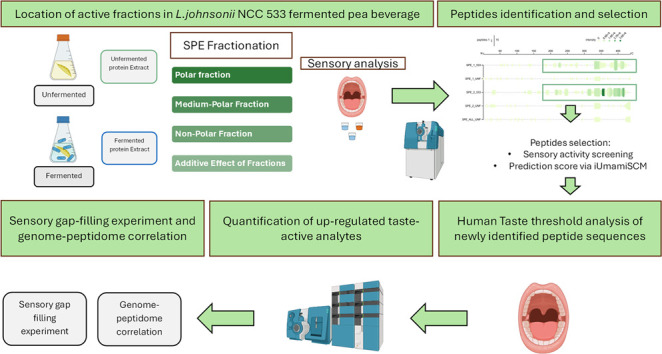

This study investigated the mechanism underlying the flavor improvement observed during fermentation of a pea protein-based beverage using *Lactobacillus johnsonii* NCC533. A combination of sensomics and sensoproteomics approach revealed that the fermentation process enriched or generated well-known basic taste ingredients, such as amino acids, nucleotides, organic acids, and dipeptides, besides six new taste-active peptide sequences that enhance kokumi and umami notes. The six new umami and kokumi enhancing peptides, with human recognition thresholds ranging from 0.046 to 0.555 mM, are produced through the degradation of *Pisum sativum*’s storage protein. Our findings suggest that compounds derived from fermentation enhance umami and kokumi sensations and reduce bitterness, thus improving the overall flavor perception of pea proteins. In addition, the analysis of intraspecific variations in the proteolytic activity of *L. johnsonii* and the genome–peptidome correlation analysis performed in this study point at cell-wall-bound proteinases such as PrtP and PrtM as the key genes necessary to initiate the flavor improving proteolytic cascade. This study provides valuable insights into the molecular mechanisms underlying the flavor improvement of pea protein during fermentation and identifies potential future research directions. The results highlight the importance of combining fermentation and senso(proteo)mics techniques in developing tastier and more palatable plant-based protein products.

## Introduction

In recent years, the use of novel pea protein-based food has garnered significant attention owing to its ethical and environmental benefits over traditional animal-based products.^1^ However, the use of pea protein has been limited due to its undesirable aroma and taste attributes, generally described as green, beany, bitter, and astringent.^2,3^ Efforts to address this sensory challenge are ongoing and have been presented in a perspective by Mittermeier et al. (2021).^4^

Among the various processing and biotechnological strategies that have been explored to optimize the flavor code of foodstuffs bearing plant-based proteins, fermentation has emerged as a particularly promising method.^5^ For example, several studies have reported that the fermentation of pea protein-based beverages using lactic acid bacteria (such as *L. plantarum* and *L. fermentum, L. acidophilus, S. thermophilus*) significantly reduces off-flavors and increases desirable aroma and taste characteristics.^6−9^ These benefits are not limited to pea-based ingredients but also extend to other plant-proteins such as oat-, sunflower- and faba-bean-based beverages.^10^ Furthermore, fermentation alters the properties of pea protein-based emulsions and induces enzymatic protein hydrolysis, a change in texture (gel formation), and a shift in pH.^7,8^ Despite these improvements, the molecular mechanisms underlying the taste enhancement of pea-protein-based food products through fermentation remain poorly understood.

In a recent study^11^ we comprehensively assessed microbial cultures involved in the fermentation of a pea protein-based beverage. Among the various strains examined, *Lactobacillus johnsonii* NCC533 was identified as a promising candidate.^11^ This microbe showed a high proteolytic activity and formed several peptides from *Pisum sativum*’s storage protein. At the same time, *Lactobacillus johnsonii* NCC533 significantly enhanced the perceived umami taste while mitigating the bitter off-flavor of the fermented product after 48 h of incubation. Moreover, we hypothised that during the fermentation umami and kokumi sensing peptides, enhancing the savory impression of pea-protein based beverages.^11^ Especially, umami and savory flavors represent a key driving force toward sustainable eating and possibly counter the notable bitter off-taste of plant protein.^12,13^

Recent advancements in the research field point out the fundamental role of fermentation-generated peptides in savory sensations.^14−21^ Integration of disciplines such as high-resolution mass spectrometry, proteomics, bioinformatics for data analysis, human sensory analysis, and genomic/transcriptomic data, as well as the ability to synthesize peptides and molecular docking simulations, has led to remarkable advancements in this field. This has facilitated the discovery of new peptides and emphasized the importance of proteolysis in flavor development.^22−25^

Therefore, the present study uses a *sensoproteomics* approach to characterize known taste-active metabolites, peptides, and newly discovered taste-active peptides of pea protein-based beverages fermented with *L. johnsonii* NCC 533. This approach has previously been used to study other fermented foodstuffs,^20,21^ with promising results.

In addition to identifying the chemical stimuli responsible for taste improvement, this study explored the genomic tools essential for eliciting proteolytic activity in *L. johnsonii* strains by leveraging intraspecific variations.

In conclusion, the research is significant because it reveals the fundamental factors that determine the taste improvement of pea protein-based beverages and provides a foundation for further exploring the taste enhancement of plant-based beverages through fermentation.

## Materials and Methods

### Chemicals

Methanol and acetonitrile (ACN) used for ultrahigh-performance liquid chromatography–mass spectrometry and for extraction analysis were of LC–MS grade (Honeywell, Seelze, Germany). The following chemicals were obtained commercially: ammonium acetate, formic acid, and acetic acid (Merck, Darmstadt, Germany). *L*-Amino acids, nucleotides, nucleosides, lactic acid, and sodium chloride were obtained from Sigma-Aldrich (Steinheim, Germany). Stable isotope-labeled amino acids and nucleotides were purchased from Cambridge Isotope Laboratories Inc. (Tewksbury, MA, USA). Synthetic reference peptides such as GQIEEL, GSAQEVD, EVDRLLKN, GQIEELSKN, GSSHEVD, ELTPE, AGEEDNVIS, EENVIVKV, ANAQPLQRE, REQIEEL, SREQIEEL, DKEEEQEEETSKQVQ, RG, RP, PS were purchased from GenScript (Leiden, The Netherlands), and *L*-glutathione was obtained from VWR (Radnot, United States). Media and chemicals used for the fermentation experiment were the same as those used in our previous study.^11^

### Pea Beverage Preparation

The pea beverage was prepared according to Spaccasassi et al.^11^ A pea protein suspension was prepared by mixing a pea protein isolate with deionized water. The suspension was then homogenized and preheated to 75 °C, immediately followed by UHT treatment. Sterilization of pea milk beverage by ultrahigh-temperature (UHT) treatment was performed at a 50-L scale. For this treatment, the prewarmed suspension was heated for 4 s to 143 °C at a flow rate of 30 L/h and then efficiently cooled to 4 °C. Finally, the plant protein beverage was aseptically filled into sterile 2-L plastic bottles and stored at 4 °C until use. Before fermentation, the sterilized beverage was manually homogenized. The raw material concentration in the beverage was 10% (m/v), resulting in a protein concentration of 8.4%, carbohydrate concentration of 0.3%, and fat concentration of 0.6% in the final beverage. Two beverages were obtained from this procedure, named NS85F and FYPP-80, using two different starting raw materials. Details of the raw material used in the study as well as composition of the raw material and the beverage are detailed in Table S1.

### Fermentation and Growth Analysis of Pea Beverage with *Lactobacillus Johnsonii* Strains

*L. johnsonii* strains from the Nestlé culture collection were used in this study. One *L. rhamnosus* strain NCC4007 was employed as nonproteolytic control (as reported in our previous screening study).^11^ All strains were stored as lyophilized isolated cultures under refrigerated conditions. The list of strains used is provided in Table 1. The initial activation passage (P1) was performed by dispersing the lyophilized culture in 10 mL of liquid medium. Cultures were incubated in de-Man-Rogosa-Sharpe MRS bouillon at a specific growth temperature of 40 or 37 °C, depending on the strain, for 48 h (reported in Table S2). A second activation passage (P2) was additionally performed. During this step, 1% of the P1 culture volume was used to inoculate the liquid medium to establish a P2 culture under specific growth conditions for 24 h at 40 or 37 °C depending on the strain (Table S2). When the P2 culture turned turbid (OD600 nm >1), 1% (v/v) of cultured P2 was used to inoculate the pea beverage formulations. The inoculated pea protein-based beverage formulations were incubated on a rotary shaker (Infors, Bottmingen, Switzerland) for 48 h at 130 rpm and at 40 or 37 °C, depending on the strain (Table S2), for 48 h and in static aerobic conditions. Noninoculated fermentation was performed as control by incubating the beverage for 48 h. All work was performed under sterile conditions using laminar flow and sterile pipettes.

**Table 1:** Effect of Fermentation by *Lactobacillus Johnsonii* Strains on a Pea Protein-Based Beverage^a^

**strain/sample**	**species**	**fermentation time**	**CFU/mL T0**	**CFU/mLT48**	**initial pH**	**final pH**	**texture after fermentation**
unfermented	-	-	-	-	6.8	-	-
NCC3033	*L. johnsonii*	48 h	1.83 × 10^07^	5.75 × 10^06^	6.85	6.52	liquid
NCC1584	*L. johnsonii*	48 h	9.00 × 10^06^	7.50 × 10^06^	6.83	6.49	liquid
NCC1680	*L. johnsonii*	48 h	1.68 × 10^07^	4.50 × 10^07^	6.74	6.48	liquid
NCC2680	*L. johnsonii*	48 h	1.55 × 10^06^	no count	6.8	6.66	liquid
NCC1657	*L. johnsonii*	48 h	5.00 × 10^06^	2.25 × 10^06^	6.8	6.71	liquid
NCC533	*L. johnsonii*	48 h	6.00 × 10^06^	7.25 × 10^06^	6.79	6.48	liquid
NCC4007	*L. rhamnosus*	48 h	1.05 × 10^07^	1.18 × 10^07^	6.78	6.76	liquid
unfermented		-	-	-	7.16	-	-
NCC3033	*L. johnsonii*	48 h	9.00 × 10^06^	2.00 × 10^06^	7.25	6.65	liquid
NCC1584	*L. johnsonii*	48 h	8.25 × 10^06^	6.00 × 10^07^	7.2	6.7	liquid
NCC1680	*L. johnsonii*	48 h	2.25 × 10^07^	1.88 × 10^08^	7.31	6.02	thick, gel-like
NCC2680	*L. johnsonii*	48 h	1.00 × 10^06^	2.25 × 10^07^	7.2	5.87	thick, gel-like
NCC1657	*L. johnsonii*	48 h	7.00 × 10^06^	6.50 × 10^07^	7.19	5.91	thick, gel-like
NCC533	*L. johnsonii*	48 h	4.75 × 10^06^	1.63 × 10^08^	7.23	5.87	thick, gel-like
NCC4007	*L. rhamnosus*	48 h	1.23 × 10^07^	1.18 × 10^07^	7.23	7.08	liquid

a This table summarizes the growth (CFU/mL), pH changes, and textural changes in a pea protein-based beverage fermented using various *L. Johnsonii* strains and in unfermented controls over 48 H. Initial and final CFU/mL counts, pH levels before and after fermentation, and the resulting texture (liquid or thick, gel-like) are presented for each strain, illustrating the diversity in fermentation outcomes.

### Determination of Colony-Forming Units (CFU). CFU were Determined by the Plate Serial Dilution Spotting Method

The enumeration was conducted through a serial dilution technique using a sterile 96-well microplate. In brief, 1 mL of culture samples was sequentially diluted using 0.85% NaCl (w/v), supplemented with 1 g/L of tryptone (Becton Dickinson). A series of six dilutions was prepared for each sample, and 20 μL from selected dilutions was spotted onto Petri dishes containing an appropriate MRS agar medium. Following an incubation period suitable for the strain (Table S2), colonies were enumerated to calculate Colony Forming Units (CFU) per milliliter. All experiments were performed in duplicate.

### Extraction and Separation by Mean of Solid Phase Extraction Fractionation of the Pea Beverage Extract

Pea beverage Nutralys (NS85F) fermented with *L. johnsonii* NCC533 for 48 and 24 h and unfermented NS85F were subjected to solvent extraction according to Glaeser et al. (2020).^2^ The extraction protocol was as follows: the dried proteins were extracted three times with a mixture of MeOH and H_2_O (1:1, v/v) by stirring for 30 min at room temperature, followed by filtration using a Büchner funnel (Rotilabo, 185 mm, type 111A, Carl Roth GmbH + Co. KG, Karlsruhe, Germany) and centrifugation for 5 min at 6577 RCF (Beckman Coulter, Brea, California). For a detailed breakdown of the extraction and fractionation processes, including volumes, yields from both unfermented and fermented materials, and specific concentrations of the prepared pea beverage formulations, refer to Table S3 in the Supporting Information. The filtrates were combined, freed from solvent by vacuum evaporation at 40 °C, and freeze-dried twice to obtain extractable metabolites. Three primary extracted materials were obtained: unfermented extracted pea beverage (UEPB) from the extraction of unfermented pea beverage and fermented extracted pea beverage (FEPB) at 24 and 48 h fermentation time (FEPB_24 h_ and FEPB_48 h_).

The SPE fractionation procedure described by Hald et al.^26^ was followed. An aliquot (1 g) of freeze-dried UEPB and FEPB_48 h_ was reconstituted in water (50 mL) and applied on a 10 g Chromabond C18_ec_ polypropylene cartridge (Macherey-Nagel, Düren, Germany) preconditioned with methanol (70 mL) followed by water (70 mL). After stepwise elution (75 mL), several fractions were obtained: a polar fraction (eluted in 100% water) called U1 and F1 from UEPB and FEPB_48 h_, respectively; a medium-polar fraction (eluted at 50% methanol in water; 75 mL) called U2 and F2 from UEPB and FEPB_48 h_, respectively; and a nonpolar fraction eluted in 100% methanol (75 mL), called U3 and F3 from UEPB and FEPB_48 h_, respectively. These fractions were freed from solvent by vacuum evaporation at 40 °C, reconstituted in water, lyophilized twice, and stored at −20 °C until further use for sensory analysis at natural concentrations.

### Untargeted Metabolomics Analysis of Pea Beverage and Its Fractions

#### Sample Preparation for Peptidomics and Untargeted Metabolomics

2.00 g ± 10 mg of varieties eachof pea beverages were weighed into Precellys 15 mL homogenization tubes filled with 1.4 mm ceramic beads (Bertin Technologies, Montigny-le-Bretonneux, France). Five mL portion of solvent (80% MeOH, 20% water) was added, and the tubes were cooled overnight at −20 °C. The samples were homogenized using a Precellys evolution homogenizer supplied with a Cryolys cooling module (Bertin Technologies, Montigny-le-Bretonneux, France) according to the following parameters: 6000 rpm, 3 × 30 s, 30 s pause between cycles, temperature maintained at 4 °C using liquid nitrogen. The homogenized samples were centrifuged at 3220 Relative Centrifugal Force (RCF) for 15 min using an Eppendorf centrifuge 5810 R (Eppendorf, Hamburg, Germany) at a stable temperature of 10 °C. Supernatants were filtered with a 0.45-μm Minisart RC 15 membrane filter (Sartorius AG, Gottingen, Germany), placed in a 1.5 mL liquid chromatography vial, and then directly measured by LC–MS analysis. Furthermore, a pooled sample containing an equal amount of each extract was prepared and used as a quality control (QC).^11^

#### UHPLC–TOF–MS Profiling of Samples

Metabolite analysis was performed using UPLC–TOF–MS on a Sciex TripleTOF 6600 mass spectrometer (Sciex Darmstadt, Germany) and a Shimadzu Nexera X2 system (Shimadzu, Kyoto, Japan) with an IonDrive ion source, operating in both positive and negative ESI modes. After every fifth sample, the instrument’s calibration was verified and corrected using an ESI Positive or ESI Negative Calibration Solution and a Calibrant Delivery System (Sciex Darmstadt, Germany). Metabolite separation was performed on two chromatographic columns in distinct batches. The first run, performed using reversed-phase (RP) liquid chromatography, consisted of a 100 × 2 mm, 1.7 μm Kinetex C18 column (Phenomenex, Aschaffenburg, Germany) with a gradient of 0.1% formic acid in water (A) and ACN containing 0.1% formic acid (B) at a flow rate of 0.3 mL/min with the following gradient: 0 min, 5% B; 2 min, 5% B; 18 min, 100% B; 21 min, 100% B; 22 min, 5% B; 25 min, 5% B. The second run, performed using hydrophilic interaction liquid chromatography (HILIC), consisted of an Acquity BEH amide 100 × 2 mm, 1.7 μm column (Waters Corporation, Milford, Unites states) with a gradient of 5 mM NH_4_Ac in H_2_O at pH 3 (A); 5 mM NH_4_Ac, 2% H_2_O in ACN at pH 3 (B) with a gradient of 0 min, 95% B; 2 min, 95% B; 10 min, 50% B; 12 min, 0% B; 15 min, 0% B; 15.5 min, 95% B; 20 min, 95% B. The column oven was maintained at 40 °C, and TOF–MS scanning was performed from *m*/*z* 50 to *m*/*z* 1500 for RP runs and from *m*/*z* 50 to *m*/*z* 1000 for HILIC chromatography. Positive and negative polarities were employed. MS/MS data were acquired in both data-dependent acquisition (IDA) and data-independent acquisition (SWATH). Ion spray voltage was set at 5500 eV for the positive ESI mode and −4500 eV for the negative ESI mode; the source temperature was 550 °C, nebulizing gas was set at 0.38 MPa, and heating gas was set at 0.45 MPa. The declustering potential was set to 80 V for all experiments, and the collision energy was 10 V for precursor ion scans and 35 V (including a 20 V collision energy spread) for the fragmentation in the individual SWATH windows as well as in the IDA experiments.

In IDA mode, 14 precursor ions were selected per cycle and set the switching criteria for isotope and precursor ions after three occurrences for 5 s to maximize the amount of acquired information. In SWATH mode, different parameters were used between the RP and HILIC separation runs. In RP mode, a series of 23 experiments covering a range of 50 to 1500 Da, overlapping 1 Da (25 ms accumulation time in high-sensitivity mode), were employed. In HILIC mode, 19 SWATH experiments were used to cover a range from 50 to 1000 Da with a 25 ms accumulation time per window acquired in high-sensitivity mode. Details of the SWATH windows are reported in Table S4. The sample list was randomized during the run. Twenty QC samples were run in an initial batch to equilibrate the system according to the matrix. In addition, a QC sample was inserted every fifth sample to provide a reference sample with which to detect analytical variation within the batch as well as a normalization tool as described in the literature.

#### Data Analysis and Statistical Evaluation

The UHPLC–TOF–MS data (one replicate for each fermented sample) were preprocessed using MS-DIAL software (version 4.9).^27^ MS/MS analysis and feature annotation were conducted using MSFINDER software.^28^ In MSDIAL, settings included MS1 and MS2 tolerance at 0.1 Da, a minimum peak height of 1000, a mass slice width of 0.1 Da, linear weighted average smoothing (4 scans), and a minimum peak width (5 scans). Middle QC files were used for retention time alignment, with a higher tolerance for HILIC (0.2 min) compared to RP (0.05 min) and a mass tolerance set at 0.05 Da. Peak table filtering was based on the ion presence in blank samples, with an intensity ratio threshold of 5. Normalization employed LOESS regression on the regularly injected QC intensities. Data processing and visualization were performed in R (version 4.2.3), employing ggplot2, ggpubr, and complexheatmap packages for heatmapping and plotting. Unsupervised multivariate analysis was performed using the R packages FactoMineR, Factoextra. Sciex Software Analyst, PeakView, and multiquant (Sciex, Darmstadt, Germany) were used for data quantification and chromatogram visualization.

#### ^3011^In Silico Peptide Identification

For peptide identification, MaxQuant software was used to process the wiff files (Version 1.6.6.0).^29^ The UniProt database provided FASTA files for the *Pisum sativum* storage protein. The processing parameters included an unspecific search (owing to unpredictable proteinase and peptidase production by bacteria), a minimum peptide length of 3, modifications (oxidation, acetylation), maximum peptide mass of 4600 Da, and “Sciex qTOF” as the MS setting. The deconvoluted data file was analyzed in R using similar packages, as described above. The protein search was performed by searching ″*Pisum sativum*″ and by downloading the ″*fasta*″ files. Further analysis was performed using the Peptigram tool for proteomics investigation.^30^ The data file was further analyzed in R: only the peptides with an identification score higher than 70 were retained.

### Quantification of Upregulated Metabolites

In our previous study,^11^ based on untargeted metabolomics data, qualitative comparisons were made to identify the upregulated taste-active metabolites in *L. johnsonii*-fermented and unfermented pea beverage. The metabolomics analysis in our screening study identified specific *L*-amino acids, nucleosides, arginyl peptides, and prolyl peptides as upregulated after 48 h of fermentation with *L. johnsonii* 533. Therefore, a targeted quantitative approach was employed in this study to investigate their concentration and, consequently, their role in flavor improvement.

FEPB_24 h_, FEPB_48 h_, and UEPB were dissolved in a methanol/water mixture (30:70, v/v). qNMR was used to determine the lactic acid. LC–MS/MS was used along with stable-isotope dilution analysis (SIDA) or external calibration to quantify free *L*-amino acids, nucleosides, arginyl peptides, and prolyl peptides as follows: Arginyl peptides were quantified using the method reported by Schindler et al. (2011).^31^ Prolyl peptides were quantified according to the method reported by Jünger and colleagues (2022).^20^ Amino acids and nucleosides were quantified as described by Meyer et al. (2016).^32^

### Peptide Quantification by Ultrahigh-Performance Liquid Chromatography–Tandem Mass Spectrometry (UHPLC–MS/MS)

The newly identified taste-active and taste-modulating peptides were quantitated on a 6500 mass spectrometer with an IonDrive TurboV-ion source (Sciex Darmstadt, Germany), operated in the positive ionization (ESI^+^) mode, and connected to a Shimadzu Nexera X2 system (Shimadzu, Kyoto, Japan). The spectrometer was operated in positive ionization (ESI^+^) mode using the MRM mode. Parameters were set as follows: an ion spray voltage of 5500 V, a curtain gas at 35 psi, gas 1 at 65 psi, gas 2 at 55 psi, a temperature of 450 °C, a collision-activated dissociation of −2 V, and an entrance potential of −10 V. The MS/MS parameters of the peptides were obtained using Skyline software.^33^ The MS parameters for each transition and retention time are reported in Table S5. An aliquot (1 μL) of FEPB_24 h_, FEPB_48 h_, and UEPB was injected into the LC–MS/MS system connected to a Kinetex C18 column (150 × 2.0 mm i.d., 1.7 μm; Phenomenex, Aschaffenburg, Germany) equipped with a guard column of the same type and a gradient of 0.1% formic acid at a flow rate of 0.3 mL/min with the following time intervals: 0 min, 1% B; 10 min; 40% B,13 min, 100% B; 15 min, 100% B; 17 min, 1% B; 20 min, 1% B. Column oven was set at 40 °C, and the injection volume of each sample was 1 μL. Analyst software (version 1.6.3, Sciex Darmstadt, Germany) was used for instrument control and data acquisition. MultiQuant (version 3.0.3, Sciex Darmstadt, Germany) was used for the data analysis. All peptides were mixed together in the concentration range 0.038–250 μmol/L. The calibration curves for all analyses were linear in the chosen concentration range (R^2^ > 0.98).

#### Quantitation Using ^1^H-Nuclear Magnetic Resonance Spectroscopy

Reference standards, including synthetic peptides, used for sensory and LC-MS quantitation and analysis were dissolved in D_2_O (5.0 mmol/L). Next, 600 μL of each reference solution was transferred to an NMR tube (178 × 5 mm id.; USC tubes, Bruker, Rheinstetten, Germany) and measured on a 400 MHz Avance III NMR spectrometer (Bruker). For instrument calibration, the reference amino acid l-tyrosine with a known concentration of 5.75 mmol/L was used, and data processing was performed as previously described.^34^ Data were processed with Topspin software (3.0; Bruker) and MestReNova (version 10.0.1; Mestrelab Research, Santiago de Compostela, Spain).

### Sensory Analysis

The sensory analyses were performed by 15 assessors (7 females and 8 males; aged 23–33 years) who had provided informed consent to participate in the sensory tests and had no history of known taste disorders. The panelists were trained in sensory experiments according to the procedure described by Jünger et al.^20^ Before human taste threshold analysis, sensory training for kokumi was further enhanced by presenting descending concentrations from 5 mM to 500 μM of model broth solution spiked with glutathione and using a triangle test to stimulate the attribute recognition abilities of the panelists. The sensory sessions were performed at 22–25 °C in air-conditioned sensory booths, and the light was adjusted to red to mask any visual differences between samples. To prevent cross-modal interactions with odorants, the panelists used nose clamps. Before analysis, the fractions and purified compounds were lyophilized twice and analytically confirmed to be essentially free of solvents. All reference synthetic peptides were screened with NMR and LC-qTOF-MS to determine their purity and the presence of contaminants. The panelists were advised to spit out the samples after tasting.

#### Comparative Taste Profile Analysis

**Figure 1:**
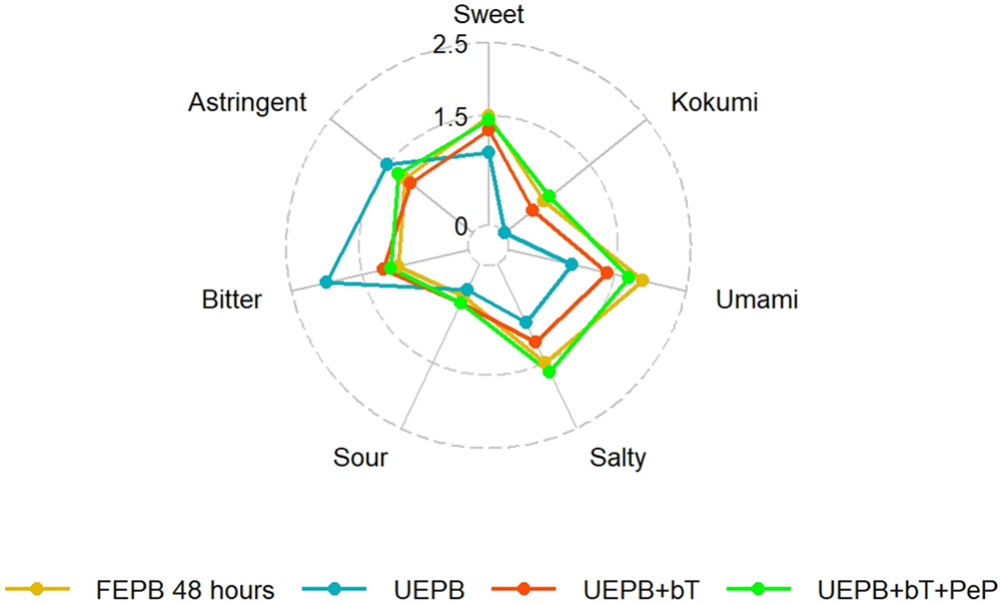
Comparative sensory profile analysis of FEPB, UEPB, UEPB+bT, and UEPB+bT+PeP. This radar chart presents a multidimensional comparison of sensory attributes including sweetness, kokumi taste, umaminess, saltiness, sourness, bitterness, and astringency for four distinct profiles: fermented extracted pea beverage (FEPB), unfermented extracted pea beverage (UEPB), unfermented extracted pea beverage with basic tastants (UEPB+bT), and unfermented extracted pea beverage with both basic tastants and peptides (UEPB+bT+PeP). Each axis represents a sensory attribute scored on a scale from 0 to 5 [only 0 to 2.5 depicted], allowing for a visual assessment of the intensity of each attribute in the respective samples. The variation in line patterns and colors facilitates comparison across samples, highlighting the taste footprint of each.

A sample (1 mL aliquot) of the reconstituted solutions of Unfermented Extracted Pea Beverage (UEPB), Unfermented Extracted Pea Beverage spiked with the basic tastants (UEPB+bT) (basic tastants motitored in this study: CMP, GMP, IMP, UMP, *L*-valine, *L*-leucine, *L*-isoleucine, *L*-phenylalanine, *L*-glutamic acid, *L*-tyrosine, *L*-histidine, *L*-lysine, *L*-arginine, *L*-alanine, *L*-proline, *L*-serine, *L*-glutamic acid, *L*-glutamine, *L*-asparagine, *L*-aspartic acid, and lactic acid^20,35^), Unfermented Extracted Pea Beverage spiked with the basic tastants and peptides UEPB+bT+PeP, and Fermented Extracted Pea Beverage for 48 h (FEPB_48 h_) were presented to the panelists. Details of the calculation of the “natural concentrations” used for UEPB and FEPB are reported in Table S3. The panelists were then asked to rate the bitterness, sweetness, sourness, umaminess, kokumi sensation, saltiness, and astringency of the samples on a scale ranging from 0 (undetectable) to 5 (strongly detectable) in comparison with the fixed scores of UEPB. The fixed scores of UEPB were obtained in a separate taste profile analysis session, where an aliquot (1 mL) of the UEPB solution at natural concentration was presented to the trained panel. The panel was asked to evaluate bitter, sweet, sour, umami, salty, and astringent taste perceptions on a scale from 0 (not detectable) to 5 (strongly detectable). This procedure allowed the fixation of values for the unfermented reference for the entire study by computing the average of the obtained scores. The results of the experiments were averaged and used to compare the different profiles, as reported in Figure 1. This approach allowed for a systematic evaluation of the effect of fermentation on the taste of pea protein-based beverages and the effect of the addition of upregulated analyte by comparing the mean values between the different samples and assessing whether the additions shifted the attributes’ values closer to the FEPB _48 h_.

#### Sensory Characterization of SPE Fraction-Based Reconstitutions

**Figure 2:**
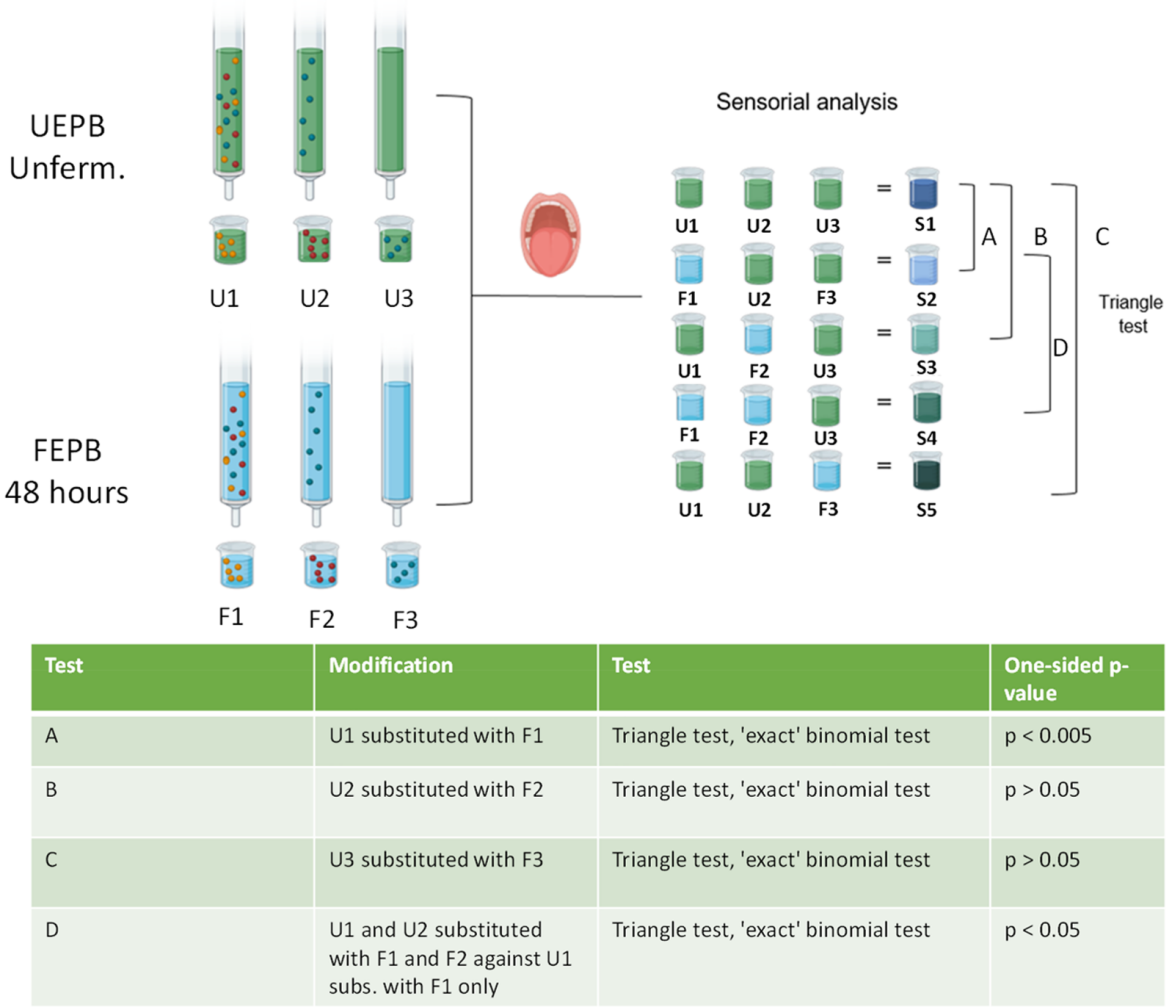
Schematic overview of solid phase extraction (SPE)-based fractionation and recombination strategies for identifying chemosensory changes in fermented products. This figure illustrates the SPE-based fractionation process and the subsequent recombination approach to identify the fractions responsible for altered chemosensory stimuli post fermentation. The table summarizes the results of triangle tests designed to detect significant sensory differences between the original and substituted fractions. The tests are labeled as Test A (U1 replaced with F1), Test B (U2 replaced with F2), Test C (U3 replaced with F3), and Test D (U1 and U2 replaced with F1 and F2 versus U1 replaced with F1 alone), with one-sided p-values from exact binomial tests provided to indicate statistical significance.

All SPE subfractions (U1, U2, U3, F1, F2, and F3) were subjected to sensory analysis after being reconstituted at a natural concentration (Table S3). First, U1, U2, and U3 were combined to obtain a reference recombinant of the full metabolome (S1). To identify the fraction with the most significant fermentation-related effect, U1, U2, and U3 were, in turn, substituted with F1, F2, and F3, generating five partial recombinants: S2 (U1 substituted with F1), S3 (U2 substituted with F2), S4 (U1 and U2 substituted with F1 and F2, respectively), and S5 (U3 substituted with F3). Recombinants S1, S2, S3, S4 and S5 were subjected to a triangle test sensory analysis. Details of the concentrations used for each solution are listed in Table S3. The statistical analysis of sensory discrimination data was performed as described in the literature.^36^ A schematic representation of this recombination experiment, the triangle tests performed, and the results obtained are presented in Figure 2.

#### Peptide Screening and Taste Activity Analysis

Active SPE fractions (F1 and F2) were assessed to identify the most promising peptide candidates using U1 and U2 as a control. Sequences of highly promising synthesized peptides were subject to *in silico* activity screening based on their intensity, fermentation origin (i.e., absent in unfermented fraction), MaxQuant identification score (>70), and predicted umami and bitter taste activity and scores estimated using iUmami SCM and iBitter SCM, respectively.^23,37^ In the activity screening phase, each peptide was tested in two solutions: Evian water (EW) adjusted to pH 5.4 with trace amounts of aqueous formic acid (1% in water) and a simplified model solution (SMS) containing concentrations of *L*-glutamic acid, lactic acid, and sodium chloride above their respective taste thresholds, also adjusted to pH 5.4.^38^ Peptides were evaluated in a sensory analysis guided by trained panelists. Three different concentrations of each peptide ([0.500, 0.050, and 0.015 mmol/L]) were tested in both the EW and SMS solutions. During the sensory analysis, each panelist was presented with six samples for each peptide. They were asked to first taste a reference solution and then taste the two solutions—one spiked with peptides and the other unspiked, and choose the one they perceived as different. If the panelists could identify a significant difference in any of the concentrations, they were asked to report the difference and the differing attributes. The peptides that were found to significantly affect the taste profile were selected for human taste threshold determination and further recombination experiment.

#### Determination of Human Taste Detection Thresholds

A protocol described by Jünger et al. (2022)^20^ was used for this analysis. To determine the intrinsic taste, trained panelists were asked to determine the taste threshold concentrations of purified synthetic peptides in the EW (adjusted to pH 5.4 with trace amounts of aqueous formic acid). To determine the taste-modulating activity of peptides, the peptides were tested in a model broth (MB) containing 2.9 g/L NaCl, 1.9 g/L monosodium glutamate, 6.4 g/L maltodextrins, and 2.1 g/L yeast extract (adjusted to pH 5.4). MB was used instead of SMS to have comparable threshold values with other work from the past.^20^ The experiment employed a duo-trio taste test protocol, using ascending concentrations of the stimulus, ranging from 0.003125 to 1 mmol/L. Panelists started the taste test at the lowest concentration and proceeded to higher concentrations sequentially; they continued this process until they could no longer detect the difference. The threshold values were calculated as the geometric mean of the individual threshold values.

#### Experiments to Fill the Sensory Gap

Two main experiments were performed in this section. The differences in analyte concentrations (FEPB_48 h_ – UEPB) were computed according to the results reported in Table S6. The difference in the analyte concentration was corrected by adding an appropriate quantity of the analytes to UEPB, which was then reconstituted at a natural concentration according to the calculation detailed in Table S3. Two main recombinant extracts were prepared: UEPB+ bT (unfermented extracted pea beverage spiked with all basic tastants to correct the concentration difference), and UEPB+bT+PeP (unfermented extracted pea beverage spiked with all basic tastants and all peptides to correct the concentration difference). These two samples were used in the sensory profile comparison to investigate the effect of the added analytes on the overall sensory profile according to the comparative taste profile detailed in the *Comparative taste profile analysis* (C–S) method section. The results obtained from the sensory profiles were averaged, and their respective profiles were compared across groups.

### Genome–Peptidome Integration for *Lactobacillus Johnsonii* Species

The acquisition of GenInfo Identifier (GI) numbers associated with proteolysis-related genes was performed through the analysis of Supporting Information provided by Liu et al.^39^ Given the obsolescence of GI numbers, a two-step conversion process was employed to retrieve the corresponding protein sequences. Initially, GI numbers were mapped to Universal Protein Resource (UniProt) Archive (UniParc) identifiers via the UniParc database (https://www.uniprot.org/uniparc/). Subsequently, these UniParc identifiers were utilized to locate and download the relevant protein sequences in FASTA format from the UniProt database (https://www.uniprot.org/). Notably, a single GI number may correspond to multiple UniProt accessions (Supplementary Table S7), potentially leading to redundant mapping in BLAST searches.

The acquired protein sequences of enzymes involved in proteolysis were subjected to BLAST analysis against the *L. johnsonii* strain genomes to investigate their presence. The selection criteria for BLAST hits included a stringent e-value threshold of 0.001 and a minimum coverage requirement of 80% relative to the query sequence. The subsequent analysis focused on identifying the most significant match (best hit) for each sequence. The data gleaned from these BLAST hits, specifically query identification and sequence identity percentages, served as the basis for constructing heatmaps.

Finally, peptidome data obtained from MaxQuant analysis (see respective section) related to the BLAST data were used to identify patterns connecting the two data sets and highlight patterns across the two data sets.

## Results and Discussion

### Identification of the Sensory and Metabolomic Changes in Pea Beverage Fermented with *Lactobacillus johnsonii* and Analysis of the Fractions

A previous study identified *L. johnsonii* NCC533 as a prominent starter strain for the fermentation of pea protein-based beverages to achieve flavor improvement.^11^ In the present study, pea beverage incubated with *L. johnsonii* for 48 h showed higher average scores for taste attributes such as umaminess (0.9 and 1.9 in UEPB and FEPB_48 h_, respectively), saltiness (0.9 and 1.5), kokumi sensation (0 and 0.7), and sweetness (1 and 1.7) and a lower average score for bitterness (2 and 1 in UEPB and FEPB_48 h_, respectively; Figure 1). This change in sensory profile is noteworthy because plant-based products often lack savory and umami traits, which is one of the current limitations of these food products.^13^

Thus, the objective of this study was to identify the key tastants developed during fermentation. First, it must be pointed out that no concentration changes were observed for the bitter and astringent compounds reported by Glaeser et al., 2020.^2^ This is consistent with our previous findings that neither the levels of fatty acids and their oxidation products nor those of detectable saponins differed among beverages fermented for 24, 48, and 72 h.^11^ Therefore, we conclude that the taste improvement is unrelated to the reduction or degradation of bitter and astringent plant metabolites.

To analyze whether previously identified taste stimuli may fill the sensory gap observed during the previous screening analysis, basic tastants were quantified in UFPB, FEPB_24 h_, and FEPB_48 h_. The list of the basic tastants upregulated during fermentation with *L. johnsonii* and their concentrations, individual taste modalities, and taste thresholds are presented in Table S6.

Subsequently, UEPB was spiked with the identified and quantified basic tastants to compensate for the concentration difference (between FEPB_48 h_ and UEPB; results are in Table S6) to form a reconstitution sample called UEPB+bT. The three extracts, UEPB, FEPB_48 h_, and UEPB+bT, were characterized by a human sensory panel using sensory profile comparison; the results are depicted in Figure 1. The sensory properties of the three samples were evaluated using seven taste attributes: sweetness, sourness, bitterness, kokumi sensation, astringency, saltiness, and umaminess. FEPB_48h_ showed the highest scores for umaminess, kokumi sensation, saltiness, and sweetness but the lowest scores for bitterness and astringency. UEPB and UEPB+bT showed lower scores for umami, kokumi, and salty attributes while lower score for bitterness than FEPB_48 h_. Moreover, FEPB_48 h_ showed the highest score for the kokumi sensation. These results suggest that the fermentation of pea beverages enhances umaminess and kokumi sensation while decreases bitterness and astringency. An effect of sweetness is also observed. The impact on umami seems to be related to the observed concentration increase in umami and umami-enchancing metabolites during the 48 h fermentation. Specifically, *L*-glutamic acid, *L*-glutamine, *L*-aspartic acid, *L*-asparagine, as well as several pyrimidine ribonucleoside 5′-monophosphates showed an increase in concentration (Table S6) over fermentation time. Increase in sweet tasting amino acids (Table S6) such as *L*-alanine, *L*-proline and *L*-serine might also be related to the increase sweet perception. Similarly, in recent literature, mono sodium glutamate (MSG) has been found as a contributor to umami taste of pea protein in subthreshold concentration, similarly to what we observe in the present study.^40^ In addition to MSG, 5′-adenosine monophosphate (AMP) and 5′-uridine monophosphate (UMP) were also found as significant contributors to umami taste of pea protein isolate, with UMP being particularly active in synergy with the others.^40^ Our results are in accordance with these data, and in addition, fermentation with *L. johnsonii* is responsible for increasing the concentration of some of these umami tasting metabolites, which consequently increase the umami sensory score (Figure 1).

The results indicate that the sensory properties of the extracted pea beverage differed in terms of bitterness, umaminess, and kokumi sensation. The addition of basic tastants to UEPB moved the respective sensory scores of UEPB+bT closer to those of FEPB; however, the sensory profile of UEPB+bT did not fully match that of FEPB, as shown in Figure 1. This suggests that some other analytes produced during fermentation are missing in UEPB+bT. Considering this finding, the sensoproteomics approach was employed to achieve complete deconvolution of additional peptides, as described previously.^20,21^

### Analysis of Fermentation-Related Chemosensory Changes

Next, we conducted a series of human sensory experiments to investigate the effect of different SPE fractions on the taste profile of pea beverages to identify the missing taste-active/taste-modulating peptides. Figure 2 is a graphical representation of the experiment and its findings, which revealed that the substitution of U1 with F1 (S2; Test A) had the most significant effect on the taste profile (*p* = 0.005). Compared with the S1 recombinant, which contained no fraction from the fermented samples, S2 showed higher scores for umaminess and kokumi taste and a lower score for bitterness. The substitution of U2 with F2 (S3; Test B) was not significant (*p* > 0.05), and the substitution of U3 with F3 in the recombinant (S5; Test C) also showed no direct effect on the overall taste profile (*p* > 0.05).

Because some panelists reported a change in mouthfeel during Test B, although the difference was not statistically significant (*p* = 0.1495), we further examined the additive effect of F1+F2+U3 against F1+U2+U3 (Test D). The experiment showed a significant difference in taste profiles (*p* < 0.05), suggesting that the sensometabolome of F1 and F2 differed from that of U1 and U2 and this change was induced by fermentation. We confirmed that substituting the unfermented polar fraction with the fermented polar fraction significantly affected the final sensory properties of the product. Additionally, substituting the medium-polar fermented fraction with the medium-polar unfermented fraction (while keeping F1 fixed instead of U1) had a significant effect (*p* < 0.05).

In summary, the sensory analysis conducted by human panelists verified that SPE fractions F1 and F2 contained modified chemosensory stimuli, a direct result of fermentation with *L. johnsonii* for 48 h. The UHPLC-ToF-MS analysis showed that many peptides are present in these fractions; therefore, we used a sensoproteomics approach to characterize the taste-active or taste-modulating peptides present in the subfractions F1 and F2.^4^ Notably, when we compared the peptide content in F1 and F2 against the unfermented controls U1 and U2, a significant increase in peptides due to the proteolytic activity of *L. johnsonii* strains was evident, supporting the findings from our previous study.^11^

**Figure 3:**
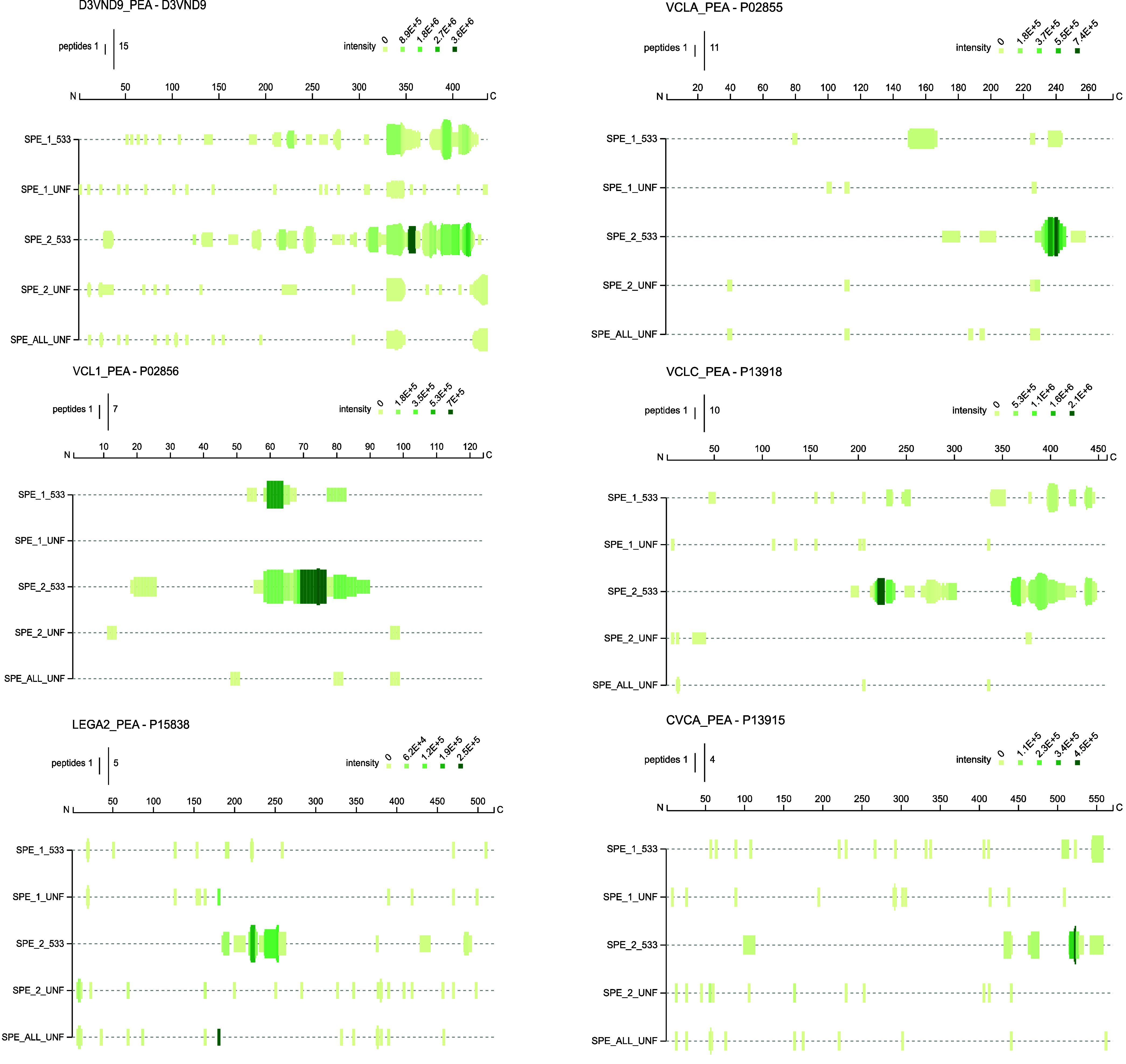
Peptide maps for each hydrolyzed protein. Maps were obtained from the downstream analysis of the data file of MaxQuant using the Peptigram software.^30^ This figure shows peptide coverage and intensity for each fermented fraction showing taste alterations and respective control. Each sample is displayed on a separate line, and a green bar is drawn for each residue position covered by at least one peptide in the sample. The height of the bar represents the number of peptides overlapping that position. The color intensity indicates the summed ion intensities of the peptides, with dark green indicating high intensity and light green indicating low intensity. SPE_1_533 is the fermented polar fraction (F1), SPE_1_UNF is the unfermented polar fraction (U1), SPE_2_533 is the fermented medium-polar fraction (F2), and SPE_2_UNF is the unfermented medium-polar fraction (U2).

Building on these observations, we conducted a detailed analysis of the fermentation-induced proteomic changes. Figure 3 shows the peptide profile of each hydrolyzed protein mapped against the protein sequence by comparing the two active fractions against the respective controls. By comparing the peptide maps and intensities over the hydrolyzed sequences between the fermented fractions and unfermented fractions, it was observed that mainly proteins D3VND9 (vicilin 47k), VCLC_PEA (vicilin), VCL1_PEA (vicilin 14 kDa component), LEGA2_PEA (legumin A2), VCLA_PEA (provicilin), CVCA_PEA (convicilin), and ALB1D_PEA (albumin-1 D) were hydrolyzed during 48 h fermentation with *L. johnsonii* NCC533. D3VND9 (vicilin 47k) was hydrolyzed the most, with 63% sequence coverage (determined by Peptigram analysis) and the highest detected relative intensity. This percentage suggests that most of the protein was hydrolyzed into smaller peptides during fermentation. Figure 3 also shows the peptide profile for each affected protein for the selected screened samples SPE_1_533 (F1), SPE_1_UNF (U1), SPE_2_533 (F2), and SPE_2_UNF (U2). In the case of the highly hydrolyzed protein vicilin 47 kDa (D3VND9_PEA), the protein’s C-terminus showed high-intensity signals for peptides in both F1 and F2. F1 and F2 profile’s slightly overlapped, indicating that peptides from this protein are present in both fractions. Conversely, the control samples without fermentation displayed significantly lower-intensity signals. These findings further confirmed that fermentation was responsible for the production of peptides in the two taste-active fractions, F1 and F2. A similar pattern was observed for other proteins, including VCLA CVCA, VCL1, and VCLC, indicating that proteolysis occurred in the fermented samples but not in the unfermented samples. These proteins also exhibited an overlapping sequence signal for the two fermented fractions, as observed for the D3VND9_PEA.

The present findings are particularly noteworthy because they identify the location and potential source of fermentation-derived taste-active peptides resulting from *L. johnsonii* proteolytic activity. Data analysis revealed a right-skewed distribution of proteolysis occurring at the C-terminus of the proteins D3VND9_PEA, CVCA_PEA, VCLC_PEA, and VCLA_PEA indicating exopeptidase activity. In contrast, in LEGA2_PEA and VCL1_PEA, proteolysis occurred predominantly in the central region (endopeptidase) of the protein sequence. Additionally, the most intense peptide signals appeared to originate from proteins acted upon by exopeptidases. D3VND9, the primary source of peptides detected, was investigated more thoroughly. Notably, the peptide sequence ANAQPLQRE characterizes the unfermented sample but is absent in the fermented sample, suggesting the proteolytic degradation of the available peptides. The sequences NAQPLQRE and AQPLQRE, which showed similar patterns, have been reported as potential sources of bitterness in pea protein-based products in recent studies employing molecular modeling. However, these studies did not confirm whether these peptides contribute to perceived bitterness *in vivo*.^41^

Furthermore, Cosson et al. (2022)^41^ found that many peptides in pea protein solutions were correlated with sensory attributes, particularly saltiness and brothy attributes, suggesting the association of certain peptides with umaminess. Some peptides were also associated with bitterness, highlighting the importance of these molecules in sensory perception. Our findings align with their conclusion that peptides play a significant role in sensory perception. Specifically, our analysis indicates that umami and kokumi attributes are heightened in fermented SPE fractions F1 and F2, which contain increased peptide levels. These results are consistent with those of Yan et al. (2021) that low-molecular-weight peptides resulting from pea protein hydrolysis are associated with more pronounced saltiness and umami enhancement, emphasizing the crucial role of protein hydrolysis in enhancing the umami, kokumi, and salty tastes.^42^ Recent literature points at fermentation-related proteolysis as well as self-digestion to be responsible for the enrichment of umami tasting peptides sequences in various foodstuffs.^14−19,25^

This finding suggests the presence of peptides derived from proteolysis that have previously been reported as taste relevant; therefore, possible taste-active peptide sequences are further characterized in the next section.

### Discovery of Novel Taste-Active Peptide Sequences via a Sensoproteomics Approach

The findings in this study indicated a high level of proteolytic activity, exhibited by the strain under investigation. Therefore, further investigation was warranted to elucidate the role of the peptides identified in the SPE fractions with taste-active and taste-modulating peptides. Specifically, the objective was to determine whether the sensory gap identified (Figure 1) may be bridged by identifying and characterizing unknown taste-active peptides present in the fractions. The findings will contribute to a better understanding of the mechanisms involved in taste perception and inform the development of strategies to enhance the taste and sensory attributes of food products.

Results obtained from the MaxQuant analysis were carefully filtered to identify peptides present only in the fermented samples (fractions F1 and F2) and absent from the unfermented samples (U1 and U2). The peptides were also assessed on the basis of their overall intensity and predicted activity using modeling tools iUmamiSCM and iBitterSCM, a protocol applied successfully in another recent study in which novel taste-active peptides were identified.^43^ The selected peptides are listed in Table S8. Peptides predicted as “umami” by the iUmamiSCM model were chosen from the F1 and F2 fractions and subjected to *in vivo* screening. These peptides included GQIEEL, GSAQEVD, EVDRLLKN, GQIEELSKN, GSSHEVD, ELTPE, AGEEDNVIS, EENVIVKV, REQIEEL, SREQIEEL, and DKEEEQEEETSKQVQ. The sequence of DKEEEQEEETSKQVQ was the longest peptide sequence identified and was predicted to exhibit the highest umami score (716.2, Table S8), making it a noteworthy target for further analysis.

Regarding bitterness prediction, GQIEEL, GSAQEVD, EVDRLLKN, GQIEELSKN, and GSSHEVD were predicted as nonbitter peptides according to the model. In contrast, AGEEDNVIS, EENVIVKV REQIEEL, SREQIEEL, ANAQPLQRE, and DKEEEQEEETSKQVQ are potential candidates imparting bitterness.

The first sensory analysis screening showed that GQIEEL, AGEEDNVIS, EVDRLLKN, GSAQEVD, GSSHEVD, and SREQIEEL were active in water (EW) in the range from 0.5 to 0.015 mmol/L. This activity was described as bitter and astringent. The sequence GQIEEL was also described as bitter and astringent; additionally, a change in the mouthfeel was reported. Notably among the five peptides predicted as non-bitter in the model prediction, four exhibited bitterness in the sensory analysis.

To verify whether the peptides have a taste-enhancing effect, all peptides were presented to a trained human sensory panel in a simplified model solution (SMS), as well. The sequences GQIEEL, AGEEDNVIS, GSAQEVD, GSSHEVD, DKEEQEETSKQVQ, ELTPE, and SREQIEEL were identified as active peptides in the tested range from 0.5 to 0.015 mmol/L. The trained panelists reported the spiked samples to have a more intense, smooth, and long-lasting mouthfeel than unspiked samples. This type of feeling is described as a kokumi sensation.^20,35^ DKEEQEEETSKQVQ and ELTPE were found to be active only in SMS and not in EW. Peptides EVDRLLKN were found to be bitter peptides in EW and not active in SMS. Substances associated with kokumi often possess little to no distinct flavor or may exhibit a slight astringent and bitter taste. Depending on the matrix, this double effect is confirmed in our study and has been observed in other studies evaluating intrinsic and taste-modulating thresholds of other taste-modulating peptides.^20,44,45^

After activity screening, the human taste thresholds were established (for the sequences that were identified as active in SMS) to determine the individual concentrations at which the peptides exhibited individual bioactivity in both MB solution and EW (Table 2). Each peptide showed a taste threshold ranging from 0.046 mmol/L (lowest kokumi threshold identified for peptide sequence DKEEQEEETSKQVQ in MB) to 0.555 mmol/L (the highest bitter astringent threshold for peptide GSSHEVD in EW). These findings introduce novel taste-active peptide sequences discovered in pea protein-based foods, contributing to emerging evidence that longer peptide sequences possess both intrinsic and taste-modulating properties. The observed taste activity in both EW and MB, along with the identified concentration range, aligns with previous findings.^20,46^ Peptides sequences identified in fermented broad bean paste presented an activity in water ranging from 0.035 to 0.386 mmol/L and modulating activity ranging from 0.051 to 0.507 mmol/L.^17^ These results are comparable to the concentration range obtained in this study. The identification of the active peptide sequences is supplemented by comprehensive MS/MS fragmentation spectra, as detailed in Figures S1, S2, S3, S4, S5, S6, and S7 in the Supporting Information, providing a robust basis for sequence verification.

**Table 2:** Threshold Concentrations of Newly Identified Taste-Active Peptide Sequences in Model Broth and Evian Water^a^

	**taste attributes**		**taste threshold**μmol/L	
**peptides sequences**	**model broth**	**Evian water**	**model broth**	**Evian water**
DKEEEQEEETSKQVQ	Kokumi	no activity	46	no activity
SREQIEEL	Kokumi, mouthfeel change	bitter/astringent	149	111
AGEEDNVIS	Kokumi, mouthfeel change	bitter/astringent	198	69
GQIEEL	Kokumi, mouthfeel change	bitter/astringent	250	223
GSAQEVD	Kokumi, mouthfeel change	bitter/astringent	189	276
GSSHEVD	Kokumi, mouthfeel change	bitter/astringent	102	552
EVDRLLKN	no activity	bitter/astringent	no activity	336

a The table shows the human taste thresholds for peptides formed during fermentation with *L. johnsonii* NCC533 for 48 h. Peptides in model broth and Evian water were examined, and their respective taste attributes (Kokumi, mouthfeel change, bitter/astringent) and threshold values (in μmol/L) are reported.

These results are not conclusive on whether these peptides sequences are important for the overall flavor of the FEPB _48 h_ yet. Therefore, researching each flavor compound under investigation within its complex domain called flavor object, particularly because synergies cannot be excluded, remains an important next step to determine what are the important sensometabolites. There is also a need to examine each taste compounds within their complex matrices at their natural conentration to establish causative relationships beyond mere correlations, acknowledging potential missing fermentation related taste actives.^35^ Subsequently, in the next section, we quantified the known and novel taste-active peptides and metabolites enhanced during fermentation with *L. johnsonii*; the results are presented in the following section. A gap-filling sensory experiment was employed to validate these findings, serving as a proof-of-concept.

### Quantitation of Taste-Active Analytes and Peptides and Gap-Filling Sensory Experiment

**Figure 4:**
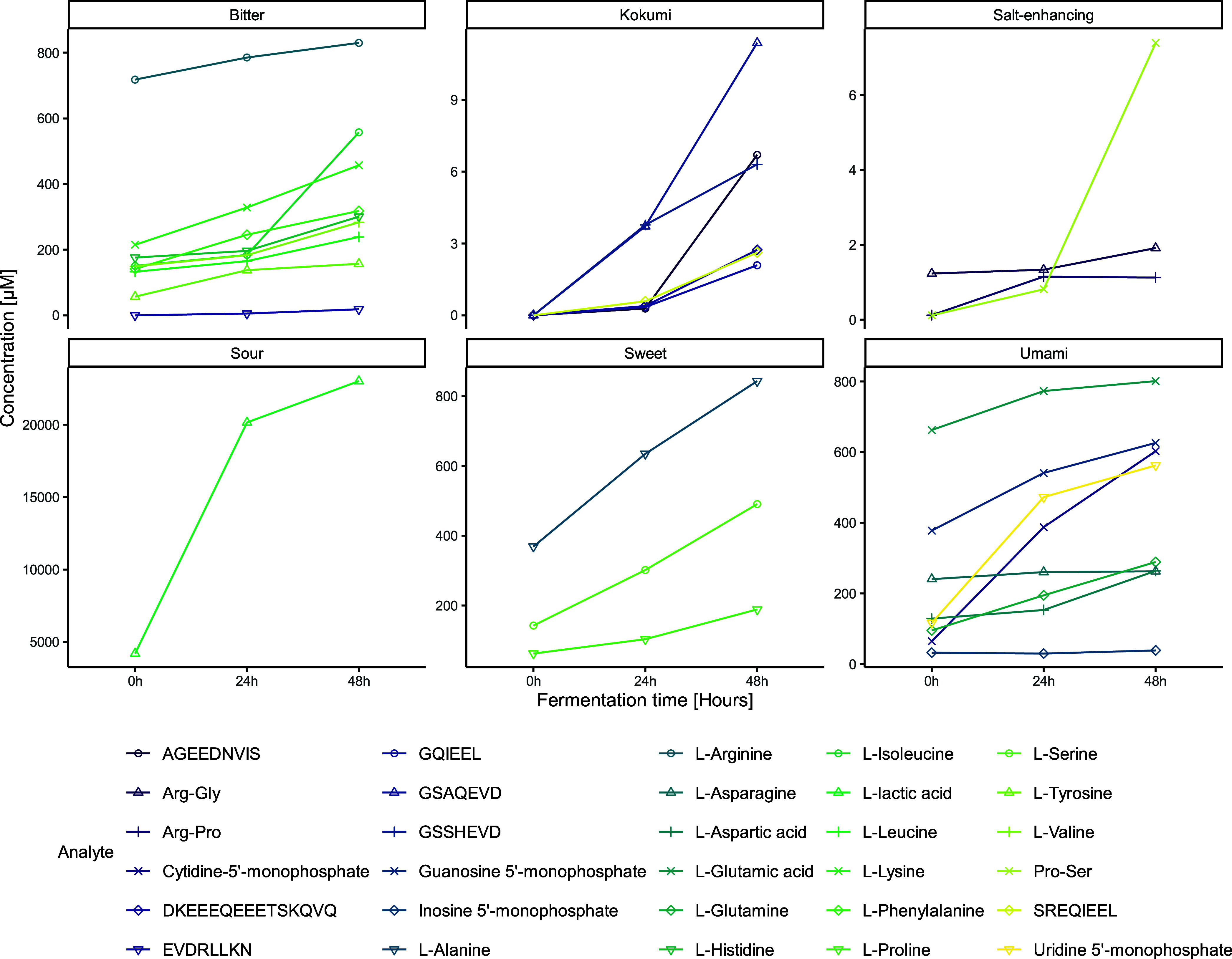
Dynamic changes in the concentration of taste-active metabolites during fermentation. This plot illustrates the variations in average concentrations (μmol/L) of different taste-active metabolites, including peptides and amino acids, across various periods of fermentation (0 h [unfermented control], 24 h, and 48 h) with *Lactobacillus johnsonii* NCC533. Metabolites are categorized by their taste properties (e.g., salt-enhancing, sweet, bitter) and visualized through both points and lines to depict trends over time. Concentrations are shown on a free-scale *y*-axis to accommodate the wide range of values, with fermentation time on the *x*-axis. Each taste category is presented in a separate panel to highlight specific changes in metabolite levels related to taste perception. The plot emphasizes the metabolic shifts that contribute to flavor development in fermented products.

As a follow-up to the previous results, the upregulated taste-active analytes developed during fermentation were quantified according to Spaccasassi et al. (2024A)^11^. Upregulated amino acids, nucleosides, Arg-Pro, Arg-Gly, and Pro-Ser as well as the newly identified longer peptide sequences described in the previous section were included in the quantification analysis. We aimed to determine each analyte’s concentration in the unfermented material and then determine how the concentration changes during fermentation. Despite its suggested activity, the peptide ELTPE was excluded from the quantification and threshold analysis because its identity could not be confirmed by comparing the retention time to the synthetic standard. This sequence was therefore classified as a false identification. Table S6 reports the average concentrations of upregulated taste-active metabolites and peptides at 0, 24, and 48 h for UEPB, FEPB_24 h_, and FEPB_48 h_. The concentrations of each taste-active metabolite and peptide were plotted against the fermentation period, categorized by taste activity (Figure 4). The results depicted in this figure indicate that fermentation with *L. johnsonii* NCC533 was responsible for the enrichment of taste-active metabolites during the 48 h fermentation period.

The selected and quantified taste-active metabolites for which an increase was observed during fermentation were chosen for a sensory gap-filling reconstitution experiment. For this proof-of-concept experiment, the concentration difference from 0 to 48 h was computed, and appropriate amounts of basic tastants and peptides were added to UEPB (UEPB+bT+PeP); then, the effect of added basic tastants and peptides was tested in a sensory profile comparison against the FEPB_48 h_ and UEPB+bT (spiked with basic tastants). The results of this experiment are shown in the spider plot in Figure 1. Adding the required concentrations of fermentation-derived analytes, including peptides and basic tastants, filled the sensory gap observed in the previous experiment. Regarding perceived umami and kokumi sensations, the sensory profile of UEPB+bT+PeP matched that of FEPB_48 h_ better than the sensory profile of UEPB+bT did. In addition, a suppressing effect on bitterness was observed. This effect was hypothesized to be related to the addition of fermentation-derived umami and umami-enhancing metabolites. These results suggest that peptides, amino acids, and nucleotides elicit umami and kokumi perceptions of the food product while decreasing the perception of bitterness. This effect can be attributed to a shift in receptor activation. Notably, this effect was observed even though all analytes, except lactic acid and Arg-Gly, were present in subthreshold concentrations. In this specific study longer peptides sequences belong to *Pisum sativum* storage protein have been idetified. Recent literature has identified taste active peptides originating from *Tetragenococcus halophilus* and *Aspergillus oryzae* fermenting soy-based products or broad beans, suggesting that also the starter culture is relevant to taste-active peptides production.^17,18^ However, in the present work the identified peptides from *Pisum sativum’* protein were enough to reproduce the desired falvor effect of fermentation with *L. johnsonii* at an incubation time of 48 h.

Given the complexity of peptide-induced activation, it seems that a taste–taste interaction occurred. Bitter–umami taste interaction is already well-known.^47^ In particular, Kim et al. (2015) found that taste-active dipeptides blocked up to 70.3% of the salicin-induced increase in Ca^2+^ influx on hTAS2R16-expressing cells. Regarding amino acids, Asp and Glu were reported to be effective in reducing the bitterness of solutions containing low concentrations of bitter amino acids.^47^ Moreover, Glu-enriched protein hydrolysates, especially acidic oligopeptides, have been associated with suppressing the bitter taste of bitter substances.^48^ These results are consistent with the present findings.

Fu et al. (2018) found that hydrolysis caused by protease A, protease P, and ProteAX after 5 h contributes to the reduced bitterness of protein hydrolysates.^49^ In their study, the enhanced umami taste was attributed to the activity of exopeptidases that cause further degradation of bitter peptides and simultaneously release smaller peptides and free amino acids; a similar biological effect was observed in the *L. johnsonii-*fermented sample. Finally, they also found that the specificity of the enzyme has a significant role in the taste of the hydrolysates.^49^ In other studies, exopeptidase treatment was reported to reduce bitterness and increase the umami and salty flavors of protein hydrolysates.^50,51^

In summary, the use of sensoproteomics to identify taste-active peptide sequences has significantly contributed to decoding the flavor stimuli associated with fermentation-related changes. However, the mechanism underlying the beneficial proteolytic activity facilitated by this bacterium remains unclear. Therefore, the genomic mechanisms underlying these metabolic activities were investigated.

### Analysis of Intraspecific Variations in the Proteolytic Activity of *Lactobacillus Johnsonii*

To explore the genomic traits responsible for the sensory improvements in pea beverages fermented with *L. johnsonii* NCC533, we analyzed the intraspecific variations in the proteolytic activity of *L. johnsonii* species using four additional strains as well as replicating the central strain of this study (NCC533), with *L. rhamnosus* NCC4007 used as a nonproteolytic negative control.^11^ Fermentation was performed on FYPP-80 and NS85F pea protein-based beverages for 48 h. The experiment (detailed in Table 1) aimed to uncover possible variations in proteolytic activity among *L. johnsonii* strains and correlate these with genomic traits. Purpose of this section is to answer whether peptide generation is strain specific. The FYPP-80 and NS85F pea protein-based beverages were fermented for 48 h, with FYPP-80 specifically chosen to check for the material’s impact.

#### Growth Analysis

**Figure 5:**
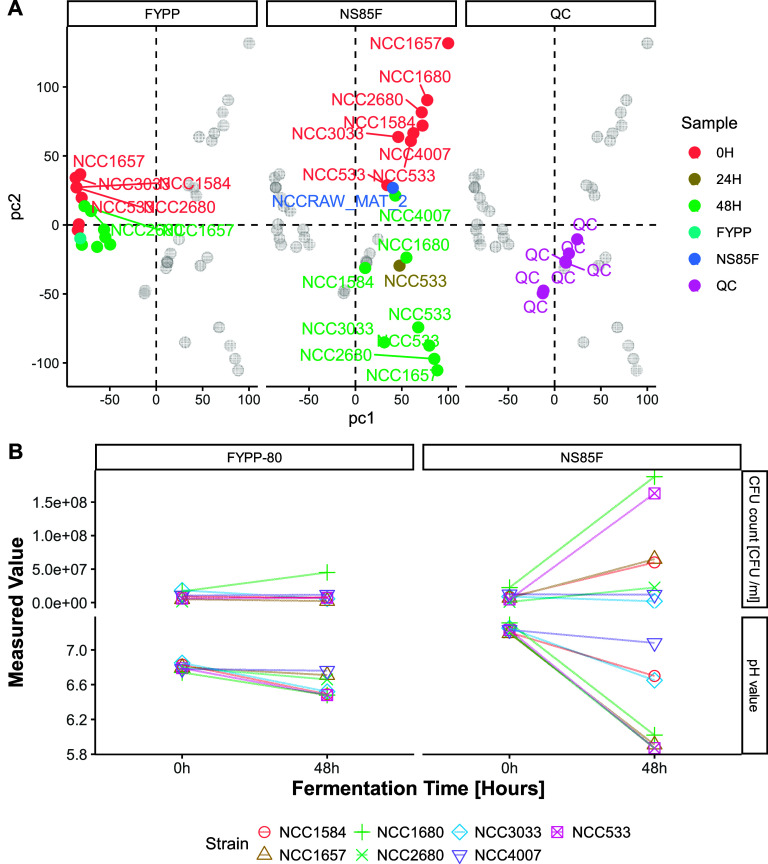
Faceted individual biplot obtained from the statistical analysis of the untargeted metabolomics alignment table via Principal Component Analysis (PCA). PCA was performed on merged tables from the different SWATH acquisitions (polarities and separation). The various colors depict the different fermentation periods and substrates. Figure B shows a scatter plot indicating values of pH and the cell count [CFU/mL] during fermentation for each strain used for the fermentation of FYPP-80 and NS85F pea protein-based beverages. The facet-grid plot is based on the material employed and the type of analysis. Lines are connecting values at 0 h fermentation time with 48 h fermentation time, grouped by strain.

Growth analysis was used to assess the ability of the strains to adapt and proliferate within the matrices. One of the key observations was the lower pH in fermented products, especially in NS85F, indicating elevated metabolic activity (Figure 5B). Strains NCC533, NCC1657, and NCC1680 significantly lowered the pH to under 5.9, causing the beverages to acquire a gel-like texture through acid gelation of pea proteins, predominantly via noncovalent interactions.^52^ In addition, we determined the cell count during the fermentation period (Figure 5B); growth is presented as the change in cell count between 0 h (control count) and the 48 h fermentation period. The figure shows partitioning based on the substrate used for fermentation. *L. johnsonii* NCC533 and NCC1680 expressed the highest growth capacity in NS85F, reaching a cell count of 1.5 × 10^8^ at 48 h. Strains NCC1584 and NCC1657 also showed growth, but to a lower extent. NCC2680 exhibited a lower growth than the other strains, whereas NCC4007 showed lower or no growth. These results showed a partial correlation to the pH results. The fastest-growing strains (NCC533 and NCC1680) showed the most significant pH-lowering capabilities. Strain NCC1657 exhibited pH-lowering capabilities as extensive as those of the other strains; however, this was not reflected in the cell count. The NCC1584 and NCC1657 strains did not show a significant pH decrease or cell count increase. In FYPP-based beverages, overall, the lower decrease in pH matched the lower increase in cell count, indicating that the product is not suitable for the growth of these bacterial strains. The only strain that showed growth in FYPP-80 was NCC1680, which also showed the highest pH decrease in FYPP80. Strains NCC3033 and NCC533 also showed a decrease in pH but did not exhibit an increase in the cell count in FYPP80.

#### Metabolomics Analysis

For the metabolomics analysis (illustrated in Figure 5A), principal component analysis (PCA) was used to distinguish between fermented and control samples with a clear separation observed between NS85F and FYPP samples. The PCA plot demonstrated more significant metabolic shifts in NS85F samples after 48 h of fermentation, consistent with changes in pH and cell counts, underscoring the effectiveness of untargeted metabolomics in capturing strain-specific and material-specific metabolic changes during pea beverage fermentation. FYPP did not seem an appropriate substrate for *L. johnsonii* fermentation, indicating a possible lack of essential nutrient. *Lactobacillus johnsonii* NCC 533 lacks the genetic machinery for amino acid and cofactor production, necessitating a dependence on external sources for these nutrients. It employs specialized transport systems, such as amino acid permeases and peptidases, to compensate for this deficiency.^53^ This reliance suggests a potential reason for its limited proteolytic activity in FYPP-based beverages, possibly due to the reduced protein accessibility from the FYPP material. Moreover, while it has the genes for synthesizing pyrimidine nucleotides (dTMP, UMP, CMP), it is missing most genes needed for purine nucleotide synthesis, retaining only those for converting existing purines to IMP, GMP, and AMP.^53^ This lack of purine nucleotides could also contribute to the observed growth limitations.

#### Genome-Peptidome Correlation

**Figure 6:**
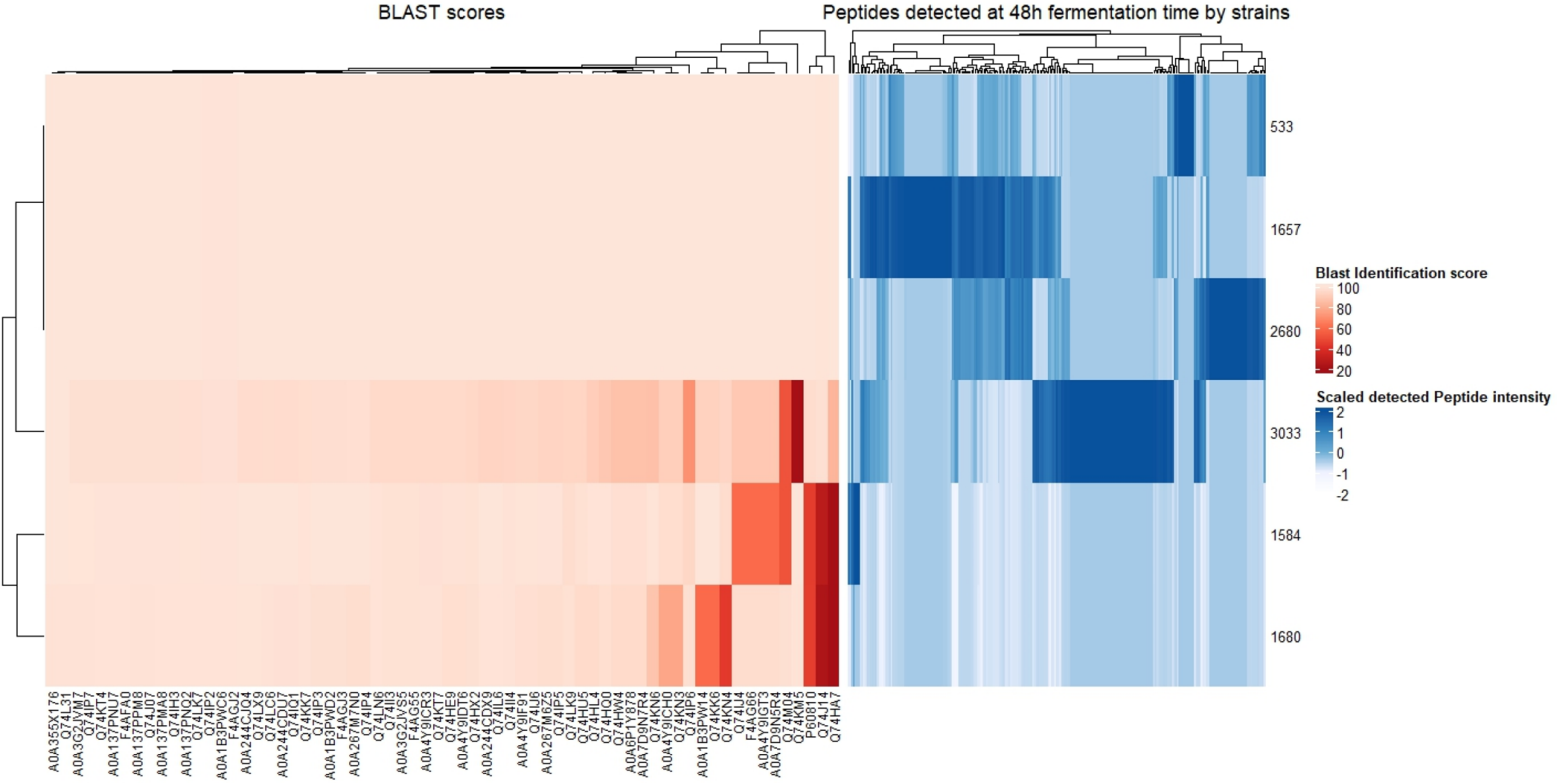
Genome–peptidome heatmap comparison. The red-colored heatmap shows the BLAST identification score matrix, which indicates the likelihood of the presence of a certain gene in the bacterial genome under investigation. Low BLAST identification scores are shown with a higher intensity of red, whereas higher BLAST scores are shown with a lower intensity of red. The blue-colored heatmap shows the fully identified peptidome and related peak areas.

Regarding the strain diverse metabolic response, this analysis not only confirmed the metabolic diversity among *Lactobacillus johnsonii* strains but also suggested a potential genomic basis for the variations in their metabolic and growth abilities. Following Liu et al. (2010),^39^ a BLAST search was performed for proteolytic genes; the analysis revealed that the strains showing lower growth and metabolic activity lacked specific genes (Tables S7 and S9). Further analysis with MaxQuant identified significant differences in peptide production among strains, with NCC533, NCC2680, NCC1657, and NCC3033 showing higher proteolytic activity, as observed in the peptidome heatmap (Figure 6). In this section, only NS85F fermented samples are included due to the lack of growth and proteolytic activity in the FYPP material observed in the previous two subsections.

The BLAST analysis of proteolytic genes revealed distinct patterns between proteolytic and nonproteolytic strains, further categorizing the differential genes into five groups: proteinases (PtrP and PtrM; UniProt IDs P60810 and Q74HA7), oligopeptide transporter system (OppA; IDs Q74IJ4, F4AG66, A0A4Y9IGT3, A0A7D9N5R4), and peptidases (PepC [IDs Q74KN6, A0A4Y9ICH0, Q74KN3], PepD [IDs A0A1B3PW14, Q74KK6, Q74KN4], and PepO [IDs Q74M04, Q74J14]).^39^ The gene profiles of strains NCC1657, NCC2660, and NCC3033, exhibiting high peptide production, were similar to those of NCC533, unlike low-peptide-producing strains, which lacked PrtP and showed variance in PrtM. Specifically, NCC1680 mirrored NCC533 in OppA genes but diverged in PepC, PepD, and PepO genes, whereas NCC1584 differed from NCC533 in the OppA and PepO genes. The gene redundancies regarding PepC, PepD, and PepO suggest overlapping functions among these enzymes. Figure 6 illustrates these genomic distinctions through heatmap comparisons, indicating that nonpeptide-producing strains lacked specific genes.

Bacterial growth in pea protein beverages is notably influenced by the presence of cell-wall-bound proteinases such as PrtP and PrtM. These enzymes play a pivotal role in lactic acid fermentation by breaking down pea storage protein into free amino acids and nitrogenous components essential for bacterial proliferation.^54^ The absence of these initial proteolytic steps in nonpeptide-producing strains hinders subsequent proteolytic cascades involving endopeptidases and aminopeptidases, which are crucial for flavor enhancement during fermentation. The transport of vicilin-derived peptides into bacterial cells is facilitated by systems such as Opp, DtpT, and Dpp, and the ATP-binding cassette transporters in the Opp system play a key role in peptide translocation across the cell membrane.^54^ Pea protein recognition by lactic acid bacteria remains a relatively new field of research compared with similar research on the milk protein casein.

## Conclusion

This study demonstrated that fermenting pea protein-based beverages with *Lactobacillus johnsonii* NCC533 enhances umami and kokumi sensations while reducing bitterness. Using a sensoproteomics approach, this study compared fermented beverages with unfermented controls and identified an enrichment in taste-active metabolites and peptides, including amino acids, nucleotides, lactic acid, and dipeptides as well as six novel kokumi/umami-enhancing peptides derived from *Pisum sativum* vicilin protein degradation. Sensory experiments confirmed that the addition of these fermentation-derived compounds to the unfermented beverage could replicate the fermented product’s sensory profile, highlighting the potential of fermentation to enhance the flavor of pea protein-based beverages by enhancing savory taste while reducing bitterness. These findings align with the previously reported flavor-modulating effects of peptides. The study also used a sensoproteomics approach to explore the metabolic changes occurring during fermentation, identifying specific proteolytic enzymes associated with flavor improvement through genome annotation and BLAST analysis.

In summary, the results highlight the importance of combining fermentation and senso(proteo)mics techniques in finding new taste-active or -enhancing peptides and in developing tastier and more palatable plant-based protein products, such as fermented pea beverages.^45^ Establishing a mechanistic understanding of the taste enhancement achieved through fermentation of pea protein beverage with *L. johnsonii* NCC 533 may provide valuable insights into improve the efficacy of reverse food engineering techniques.

## Supporting Information Available

Tandem mass spectrometry (MS/MS) fragmentation spectra of the identified peptides (Figure S1-Figure S7), Composition of the raw materials and pea beverages (Table S1), details of the strains and incubation conditions (Table S2), Extraction and SPE yelds (Table S3), SWATH-MS parameters (Table S4), MRM transitions and retention time for peptides quantitation (Table S5), Quantitation results (Table S6), Set of genes used for BLAST analysis (Table S7), Umami and bitter score predictions (Table S8), BLAST results (Table S9) (PDF)
