# Supplementary material for: Sensoproteomic Characterization of Lactobacillus Johnsonii-Fermented Pea Protein-Based Beverage: A Promising Strategy for Enhancing Umami and Kokumi Sensations while Mitigating Bitterness
Source: J Agric Food Chem. 2024 Jul 3;72(28):15875–89. doi: 10.1021/acs.jafc.4c02317 (PMC11261612; doi:10.1021/acs.jafc.4c02317)
Supplement: Supplementary file 1 — jf4c02317_si_001.pdf [file jf4c02317_si_001.pdf]

Supplementary Materials

Sensoproteomics characterization of *Lactobacillus johnsonii* fermented pea protein-based beverage: a promising strategy for enhancing umami and kokumi sensations while mitigating bitterness

Andrea Spaccasassi[†*], Lijuan Ye[#], Christian Rincon[#], Rosa Aragao Börner[#], Biljana Bogicevic[#], Arne Glabasnia[#], Thomas Hofmann[†], and Corinna Dawid[^†*]

[†] Chair of Food Chemistry and Molecular and Sensory Science, TUM School of Life Sciences, Technical University of Munich, Lise-Meitner-Str. 34, 85354 Freising, Germany

* TUM CREATE, 1 CREATE Way, #10-02 CREATE Tower, Singapore, 138602 Singapore

Société des Produits Nestlé S.A., Nestlé Research, Route du Jorat 57, CH 1000 Lausanne 26, Switzerland

^ Professorship for Functional Phytometabolomics, TUM School of Life Sciences, Technical University of Munich, Lise-Meitner-Str. 34, 85354 Freising, Germany

DKEEEQEEETSKQVQ

m/z 612.61 [M+3H]3+

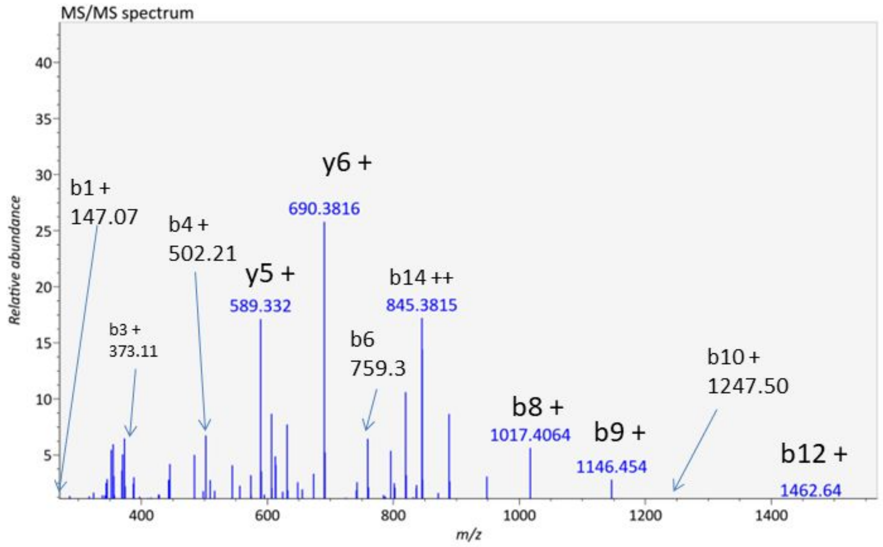

Figure S1: Tandem mass spectrometry (MS/MS) fragmentation spectrum of the peptide with sequence DKEEEQEEETSKQVQ. The spectrum displays the relative abundance of detected ions versus the mass-to-charge ratio (m/z). Annotation of b- and y-type fragment ions is indicated, corresponding to the cleavage of peptide bonds. The precursor ion is indicated with a m/z of 612.61 [M+3H]3+, suggesting a triply charged state.

AGEENDNVIS
m/z 524.23 [M+2H]2+

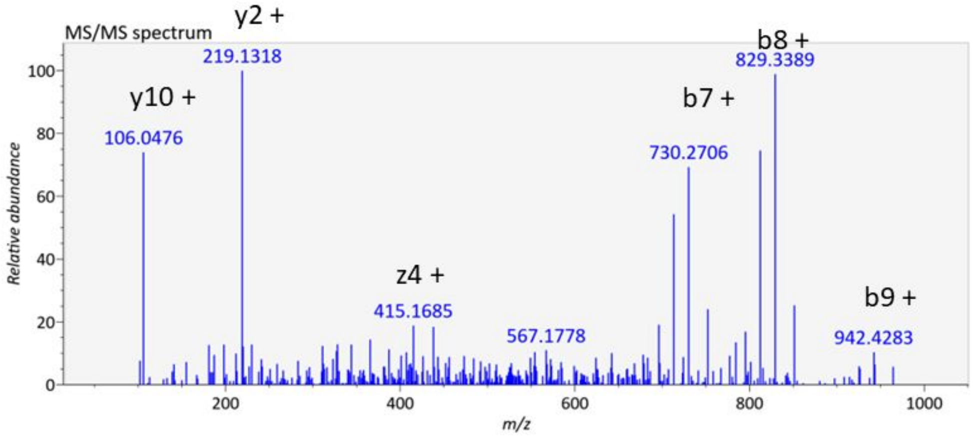

Figure S2: Tandem mass spectrometry (MS/MS) fragmentation spectrum of the peptide with sequence AGEENDNVIS. The spectrum displays the relative abundance of detected ions versus the mass-to-charge ratio (m/z). Annotation of b-, z- and y-type fragment ions is indicated, corresponding to the cleavage of peptide bonds. The precursor ion is indicated with a m/z of 612.61 [M+2H]2+, suggesting a double charged state.

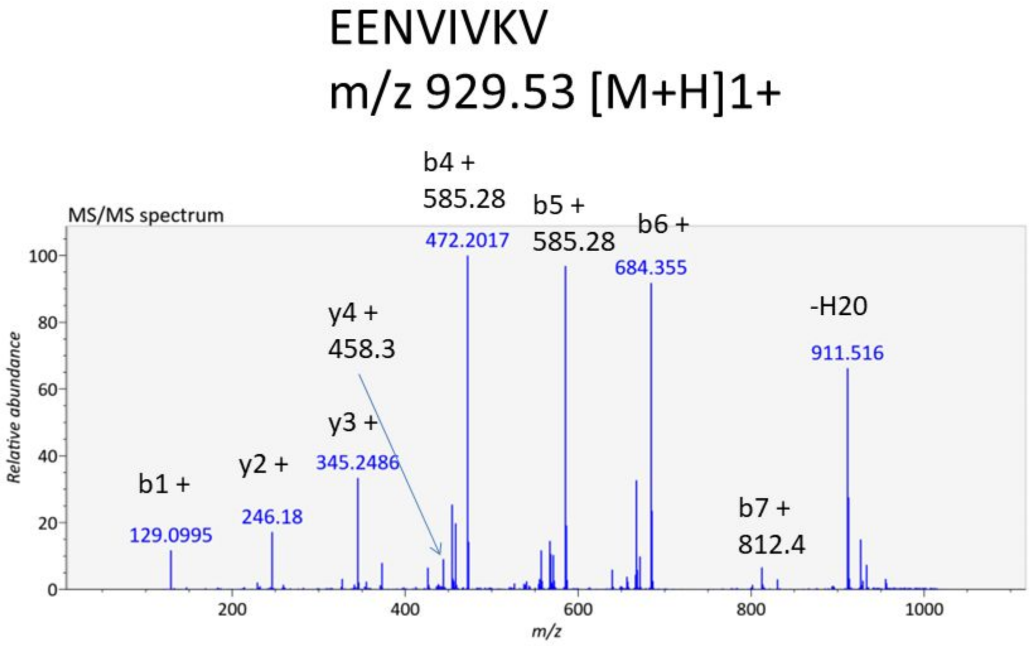

Figure S3: Tandem mass spectrometry (MS/MS) fragmentation spectrum of the peptide with sequence EENVIVKV. The spectrum displays the relative abundance of detected ions versus the mass-to-charge ratio (m/z). Annotation of b- and y-type fragment ions is indicated, corresponding to the cleavage of peptide bonds. The precursor ion is indicated with a m/z of 929.53 [M+1H]1+, suggesting a single charged state.

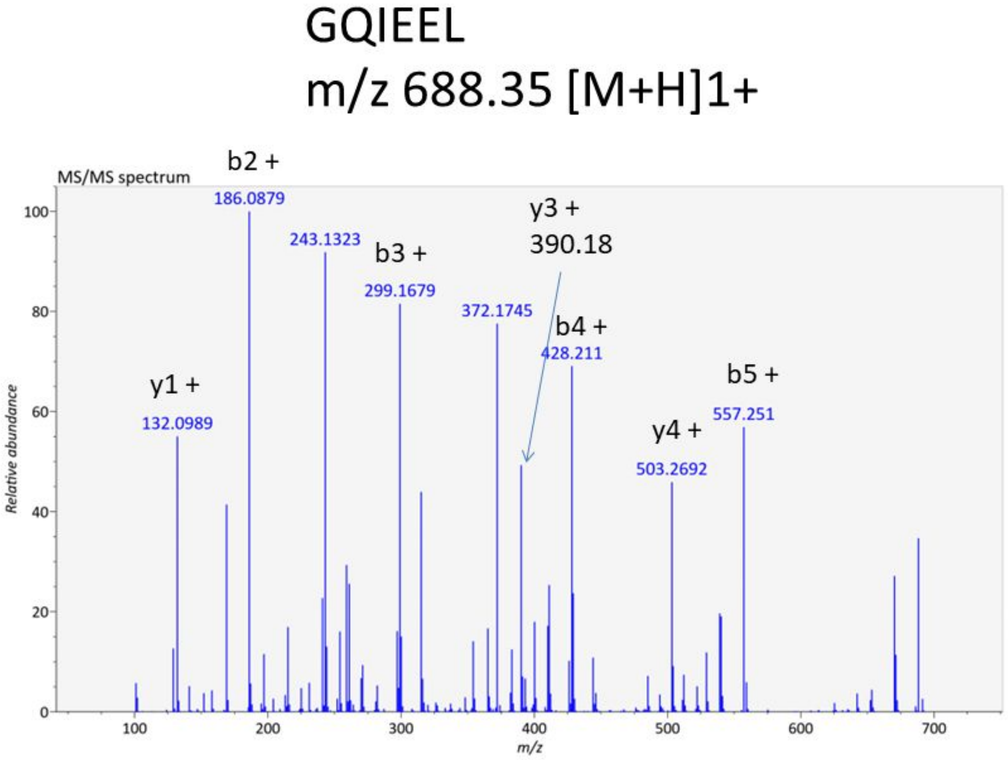

Figure S4: Tandem mass spectrometry (MS/MS) fragmentation spectrum of the peptide with sequence GQIEEL. The spectrum displays the relative abundance of detected ions versus the mass-to-charge ratio (m/z). Annotation of b- and y-type fragment ions is indicated, corresponding to the cleavage of peptide bonds. The precursor ion is indicated with a m/z of 688.35 [M+1H]1+, suggesting a single charged state.

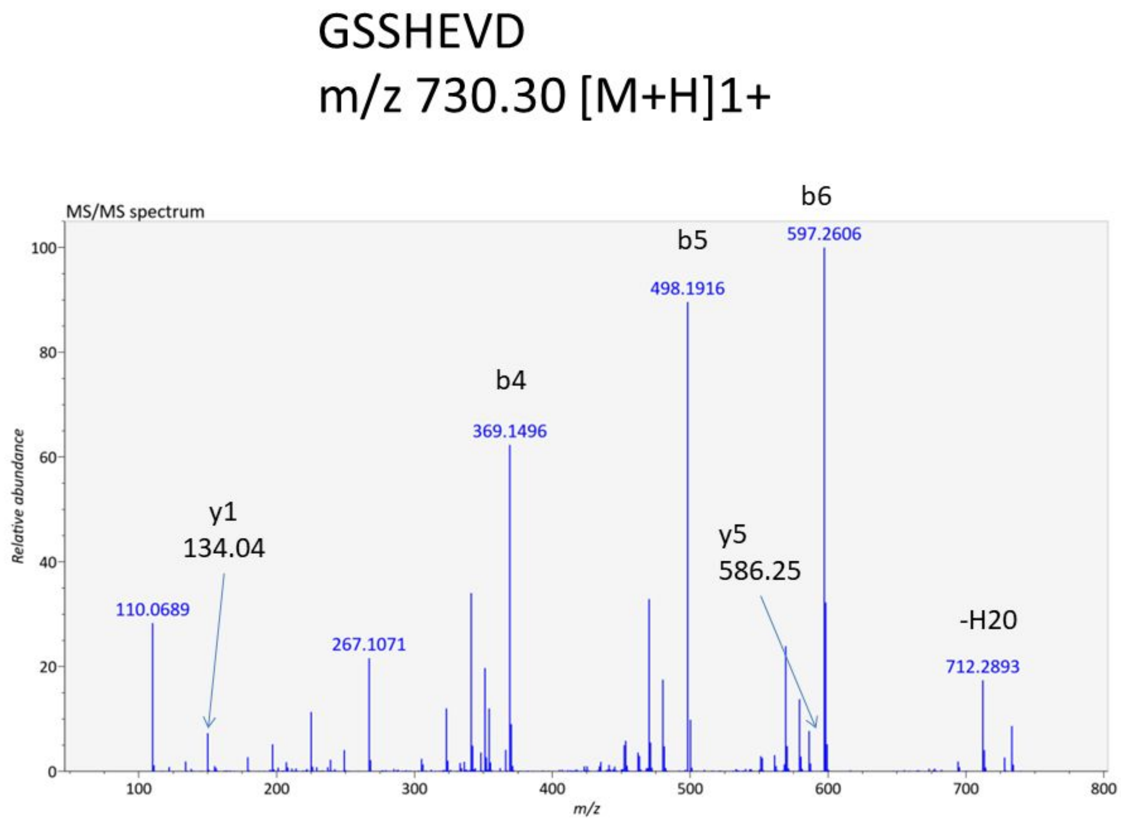

Figure S5: Tandem mass spectrometry (MS/MS) fragmentation spectrum of the peptide with sequence GSSHEVD. The spectrum displays the relative abundance of detected ions versus the mass-to-charge ratio (m/z). Annotation of b- and y-type fragment ions is indicated, corresponding to the cleavage of peptide bonds. The precursor ion is indicated with a m/z of 730.30 [M+1H]1+, suggesting a single charged state.

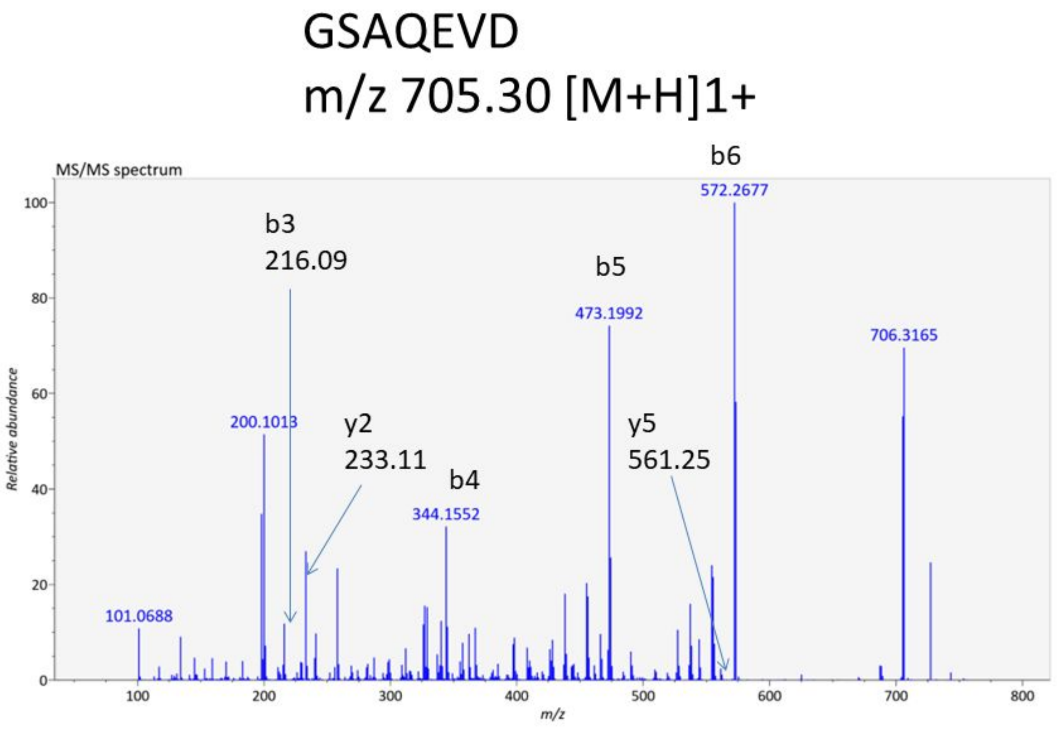

Figure S6: Tandem mass spectrometry (MS/MS) fragmentation spectrum of the peptide with sequence GSAQEVD. The spectrum displays the relative abundance of detected ions versus the mass-to-charge ratio (m/z). Annotation of b- and y-type fragment ions is indicated, corresponding to the cleavage of peptide bonds. The precursor ion is indicated with a m/z of 705.30 [M+1H]1+, suggesting a single charged state.

SREQIEEL
m/z 502.25 [M+2H]2+

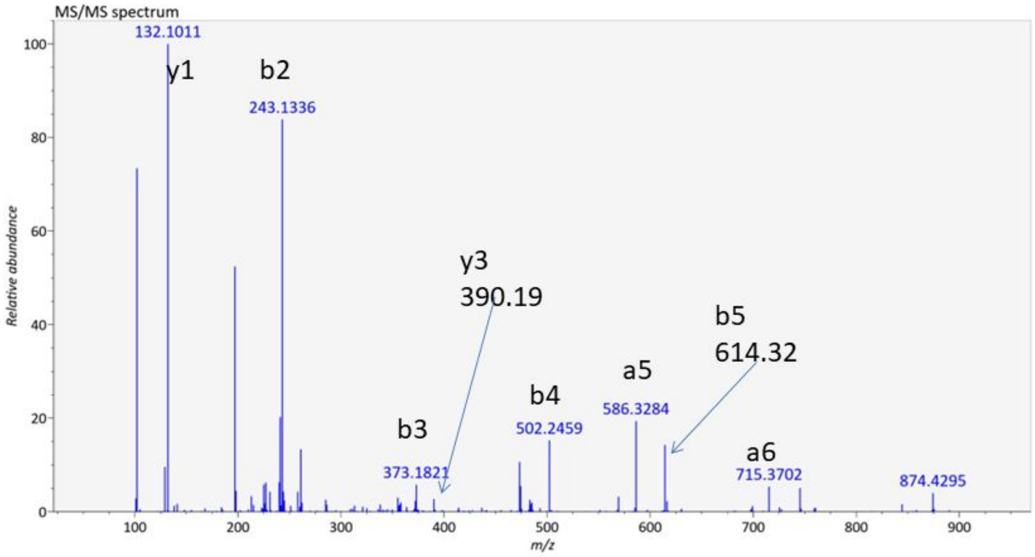

Figure S7: Tandem mass spectrometry (MS/MS) fragmentation spectrum of the peptide with sequence SREQIEEL. The spectrum displays the relative abundance of detected ions versus the mass-to-charge ratio (m/z). Annotation of b- and y-type fragment ions is indicated, corresponding to the cleavage of peptide bonds. The precursor ion is indicated with a m/z of 705.30 [M+2H]2+, suggesting a single charged state.

Additional Tables:

Table S1. Compositions of the two raw materials employed to produce the pea beverages.

**Raw material**	**Protein Pea Isolate FYPP-80**	**Protein Pea Nutralys S85F**
Supplier	AGT	Roquette
Protein content in raw material %	81.6	84
Carbohydrate content in raw material %	3	3
Fat content in raw material %		6
Raw material concentration in beverages/100 mL	10	10
Protein content in plant beverage%	8.16	8.4
Carbo content in plant beverage %	0.3	0.3
Fat content %		0.6

Table S2. Summary of the strains employed, the materials, the P1 and P2 preculture time, the preculture OD, the inoculum (%), the growth conditions, the fermentation time and the fermentation volume.

Material	NCC Strain	Species	Batch cryostock	Pre-culture time	Preculture OD (for tracking)	Inoculum 1% (mL)	Growth conditions	Fermentation time	Volume in plastic bottle (mL)
FYPP-80	unfermented	-	-	-	-	-	-	-	250
FYPP-80	NCC3033	*Lactobacillus johnsonii*	0EO25	P1 48h P2 24h	5.4	3	40°C / static areobic	48 h	200
FYPP-80	NCC1584	*Lactobacillus johnsonii*	LJ 17 14/2/96	P1 24h P2 24h	6.2	3	40°C / static areobic	48 h	200
FYPP-80	NCC1680	*Lactobacillus johnsonii*	0NN25	P1 24h P2 24h	6.9	3	40°C / static areobic	48 h	200
FYPP-80	NCC2680	*Lactobacillus johnsonii*	0ZI24	P1 48h P2 24h	9.9	3	40°C / static areobic	48 h	200
FYPP-80	NCC1657	*Lactobacillus johnsonii*	0HS25	P1 48h P2 24h	9	3	40°C / static areobic	48 h	200
FYPP-80	NCC533	*Lactobacillus johnsonii*	1NA18	P1 24h P2 24h	9.9	3	37°C / static areobic	48 h	200
FYPP-80	Neg crtl NCC4007	*Lactobacillus rhamnosus*	1EU14	P2 24h	8.6	3	37°C / static areobic	48h	200
NS85F	unfermented	-	-	-	-	-	-	-	250
NS85F	NCC3033	*Lactobacillus johnsonii*	0EO25	P1 48h P2 24h	5.4	3	40°C / static areobic	48 h	200
NS85F	NCC1584	*Lactobacillus johnsonii*	LJ 17 14/2/96	P1 24h P2 24h	6.2	3	40°C / static areobic	48 h	200
NS85F	NCC1680	*Lactobacillus johnsonii*	0NN25	P1 24h P2 24h	6.9	3	40°C / static areobic	48 h	200
NS85F	NCC2680	*Lactobacillus johnsonii*	0ZI24	P1 48h P2 24h	9.9	3	40°C / static areobic	48 h	150
NS85F	NCC1657	*Lactobacillus johnsonii*	0HS25	P1 48h P2 24h	9	3	40°C / static areobic	48 h	100
NS85F	NCC533	*Lactobacillus johnsonii*	1NA18	P1 24h P2 24h	9.9	3	37°C / static areobic	48 h	100

NS85F	neg ctrl NCC4007	*Lactobacillus rhamnosus*	1EU14	P2 24h	8.6	3	37°C / static areobic	48h	200

Table S3. Summary of extraction and solid phase extraction (SPE) Yields from pea beverage materials, including solvent usage, material recovery, and desired concentrations for subsequent application in pea beverage formulations

**Variable**	**Value**	**Unit**
Starting volume Pea Beverage	3400	mL
Starting material dry	340	g in 3400 mL
volume of solvent used (50%/50% MeOH/H2O)	1400	mL
Material obtained from 3 extractions	22.478	g
yield solvent extraction	6.611	%
Extracted Pea Beverage natural concentration	0.1983	g in 30 mL
10 g used for SPE fractionation	10	g
SPE 1 100 H2O	4.126	g
SPE 2 50%/50% MeOH/H2O	2.324	g
SPE 3 100%MeOH	0.8932	g
yield SPE in g	7.343	g
yield SPE in %	73.430	%
SPE 1 100 H2O yields	2.728	%
SPE 2 50%/50% MeOH/H2O yields	1.536	%
SPE 3 100%MeOH yields	0.591	%
Desired volume U1	30	mL
Desired volume U2	30	mL
Desired volume U3	30	mL
Corresponding pea beverage dry material used for SPE	151.259	g
Corresponding volume in beverage 10% used for SPE	1512.590	mL
Desired concentration of SPE1 pea beverage natural concentration	0.0818	g in 30mL
Desired concentration of SPE2 pea beverage natural concentration	0.0461	g in 30mL
Desired concentration of SPE3 pea beverage natural concentration	0.0177	g in 30mL

Table S4. SWATH method table: MS experiments conducted at each cycle time for each swath window. The upper part of the table reports the SWATH windows for the reverse phase method, whereas the lower part reports the windows for the HILIC method. The HILIC method is acquired up from 50 to 1000 Da, whereas the reversed-phase method is acquired up to 1500 Da.

**experiments REVERSED PHASE METHOD**	**MS Type**	**Min *m/z***	**Max *m/z***
0	TOF SCAN	50	1500
1	SWATH	49.5	96
2	SWATH	95	131
3	SWATH	130	159.6
4	SWATH	158.6	190.1
5	SWATH	189.1	215.6
6	SWATH	214.6	243.6
7	SWATH	242.6	274.1
8	SWATH	273.1	303.6
9	SWATH	302.6	341.2
10	SWATH	340.2	374.6
11	SWATH	373.6	421.8
12	SWATH	420.8	453.2
13	SWATH	452.2	482.8
14	SWATH	481.8	513.8
15	SWATH	512.8	543.8
16	SWATH	542.8	593.1
17	SWATH	592.1	652.8
18	SWATH	651.8	705.3
19	SWATH	704.3	806.5
20	SWATH	805.5	1000.5
21	SWATH	999.5	1100.5
22	SWATH	1099.5	1200.5
23	SWATH	1199.5	1500
Experiments HILIC METHOD	**MS TYPE**	**MIN *m/z***	**MAX *m/z***
0	SCAN	49.5	1000
1	SWATH	49.5	135.5
2	SWATH	134.5	186
3	SWATH	185	222.5
4	SWATH	221.5	247.5
5	SWATH	246.5	267.5
6	SWATH	266.5	292.5
7	SWATH	291.5	315.5
8	SWATH	314.5	339.5
9	SWATH	338.5	365
10	SWATH	364	387.5

11	SWATH	386.5	421.5
12	SWATH	420.5	462.5
13	SWATH	461.5	493.5
14	SWATH	492.5	524
15	SWATH	523	562.5
16	SWATH	561.5	617.5
17	SWATH	616.5	669.5
18	SWATH	668.5	725.5
19	SWATH	724.5	815.5
20	SWATH	814.5	1000.5

Table S5. MRM transitions used for quantitation of long-chain peptides employing LC-MS/MS analysis, retention time (Rt, [min]), quantifier/qualifier Q1/Q3, declustering potential (DP, [V]), entrance potential (EP, [V]) collision energy (CE) and CXP (collision cell exit potential). Common settings for all peptides: EP 10V, DP 80V, Dwell time 15 ms.

Peptide	Q1 / Q3	Rt [min]	DP [V]	EP, [V]	CE [V]	CXP [V]
EENVIVKV (Q)	465.3 / 458.3	7.5	80	10	21.8	7.5
EENVIVKV	465.3 / 246.2	7.5	80	10	21.8	7.5
REQIEEL (Q)	458.7 / 656.3	6.9	80	10	21.5	6.9
REQIEEL	458.7 / 132.1	6.9	80	10	21.5	6.9
SREQIEEL (Q)	502.3 / 743.4	6.9	80	10	23.6	6.9
SREQIEEL	502.3 / 872.4	6.9	80	10	23.6	6.9
DKEEEQEEETSKQVQ (Q)	612.6 / 147.1	5.2	80	10	27.4	5.2
DKEEEQEEETSKQVQ	612.6 / 690.4	5.2	80	10	27.4	5.2
ANAQPLQRE (Q)	513.8 / 642.4	5.4	80	10	24.2	5.4
ANAQPLQRE	513.8 / 186.1	5.4	80	10	24.2	5.4
GQIEEL (Q)	688.4 / 428.2	7.35	80	10	32.7	7.35
GQIEEL	688.4 / 557.3	7.35	80	10	32.7	7.35
GSAQEVD (Q)	705.3 / 572.3	4.4	80	10	33.6	4.4

GSAQEVD	705.3 / 473.2	4.4	80	10	33.6	4.4
EVDRLLKN (Q)	329.5 / 261.2	6.1	80	10	13.8	6.1
EVDRLLKN	329.5 / 374.2	6.1	80	10	13.8	6.1
GQIEELSKN (Q)	509.3 / 186.1	6	80	10	24	6
GQIEELSKN	509.3 / 719.4	6	80	10	24	6
GSSHEVD (Q)	365.7 / 134.0	2.9	80	10	16.9	2.9
GSSHEVD	365.7 / 597.3	2.9	80	10	16.9	2.9
ELTPE (Q)	588.3 / 245.1	5.9	80	10	27.8	5.9
ELTPE	588.3 / 346.2	5.9	80	10	27.8	5.9
AGEEDNVIS (Q)	933.4 / 715.3	6.9	80	10	44.7	6.9
AGEEDNVIS	933.4 / 828.4	6.9	80	10	44.7	6.9

Table S6. Quantitation results of the upregulated metabolites in the extracted pea beverage fermented at 0 h (UEPB), fermented for 24 h (FEPB $_{24\ hours}$) and fermented for 48 h (FEPB $_{48\ hours}$). The table reports each analyte's concentration at 0h, 24h, and 48h. Samples were run in triplicates, and the mean concentration was reported in µmol/L with Relative Standard Deviations (RSD [%]).

			**Concentration [µmol/L] (RSD [%])**		
**Compound and threshold literature source**	**Category**	**Threshold [µmol/L]**	**UEPB**	**FEPB $_{24\ hours}$**	**FEPB $_{48\ hours}$**
**Bitter**					
L-Valine [a]	Basic tastants	30000	149.3 (± 7.2)	183.2 (± 12.2)	283.5 (± 5.7)
L-Leucine [b]	Basic tastants	12000	132.7 (± 16.0)	164.9 (± 12.3)	238.8 (± 14.5)
L-Isoleucine [b]	Basic tastants	11000	150.5 (± 8.2)	184.0 (± 11.2)	557.5 (± 13.3)

L-Phenylalanine	Basic tastants	45000	141.2 (± 4.1)	245.3 (± 2.5)	317.9 (± 5.3)
L-Tyrosine [b]	Basic tastants	5000	56.8 (± 8.8)	137.6 (± 9.2)	156.8 (± 2.3)
L-Histidine [b]	Basic tastants	48000	176.05 (± 7.2)	196.35 (± 15.3)	300.7 (± 11.3)
L-Lysine [b]	Basic tastants	85000	214.9 (± 1.8)	328.4 (± 2.3)	457.1 (± 5.5)
L-Arginine [b]	Basic tastants	75000	718.0 (± 7.6)	785.5 (± 4.2)	830.0 (± 5.9)
Hypoxanthine	Basic tastants	9000	200.2 (± 1.1)	210.3 (± 6.8)	340.3 (± 7.1)
EVDRLLKN	Peptides	276	not detected	5.15 (± 3.3)	18.145 (± 3.6)
**Sweet**					
L-Alanine [b]	Basic tastants	8000	368.8 (± 5)	635.0 (± 4.2)	843.5 (± 11.3)
L-Proline [b]	Basic tastants	23275	61.7 (± 3.5)	102.7 (± 7.7)	188.3 (± 4.4)
L-Serine [b]	Basic tastants	40000	141.9 (± 8.3)	301.5 (± 7.2)	490.8 (± 6.3)
**Umami**					
L-Glutamic acid [a]	Basic tastants	1120	662.5 (± 9.3)	773.3 (± 3.2)	801.0 (± 1.1)
L-Glutamine [a]	Basic tastants	50000	94.9 (± 13.0)	194.3 (± 11.5)	288.7 (± 6.2)
L-Aspartic acid [a]	Basic tastants	600	128.4 (± 9.3)	153.0 (± 3.3)	263.6 (± 5.6)
L-Asparagine [a]	Basic tastants	50000	240.2 (± 21.7)	260.2 (± 16.4)	262.1 (± 11.3)
Cytidine-5'-monophosphate [d]	Basic tastants	40000	64.45 (± 1.2)	387.2 (± 13.0)	602.5 (± 9.4)

Guanosine 5'-monophosphate [d]	Basic tastants	30000	377.5 (± 6.8)	541 (± 7.1)	626.0 (± 11.3)
Uridine 5'-monophosphate [d]	Basic tastants	17000	116.5 (± 5.0)	472.4 (± 3.2)	562.5 (± 4.5)
Inosine 5'-monophosphate [d]	Basic tastants	5000	31.9 (± 2.2)	29.5 (± 3.3)	38.4 (± 7.8)
**Sour**					
L-lactic acid [d]	Basic tastants	14000	4200	20154	23010
**salt-enhancing**					
Arg-Pro [c]	Peptide	3.4	not detected	1.1 (± 23.6)	1.1 (± 12.3)
Arg-Gly [c]	Peptide	1.2	1.2 (± 21.6)	1.3 (± 5.4)	1.9 (± 15.2)
pro-Ser	Peptide	1300	0.117 (± 16.2)	0.813 (±4.3)	7.39 (± 11.2)
**Kokumi**					
GSAQEVD	Peptide	189	not detected	3.714 (±5.2)	11.375 (± 6.3)
GQIEEL	Peptide	223	not detected	0.3426 (± 7.4)	2.0885 (±5.2)
GSSHEVD	Peptide	102	not detected	3.7 (± 3.5)	6.3 (± 11.2)
AGEEDNVIS	Peptide	198	not detected	0.3 (±2.3)	6.7 (± 4.4)
SREQIEEL	Peptide	177	not detected	0.6 (± 3.6)	2.6 (± 4.5)
DKEEEQEEETSKQVQ	Peptide	46	not detected	0.4 (± 7.7)	2.7 (± 8.2)

[a] Toeslstede and Hofmann. [2]

[b] Rotzoll,Dunkel, and Hofmann [3]

[c] Schindler et al. [4]

[d] Dunkel et al., [5].

[e] Salger er al.,[6]

Table S7 Comparative Analysis of Gene Presence in *L. johnsonii* Strains as Identified by BLAST Analysis. This table lists genes compared across different strains of L. *johnsonii*, including their UniProt IDs, GI numbers, gene names, and protein categories. The data is based on research by Liu et al. (2010), employing a stringent e-value threshold of 0.001 and a minimum coverage requirement of 80% for BLAST hits. It includes various protein categories such as ABC transporter systems, aminopeptidases, cell-wall bound proteinases, di/tripeptides uptake proteins, dipeptidases, endopeptidases, oligopeptides uptake components, proline peptidases, and tripeptidases.

UniProt ID	GI number	Gene Name	Protein Category
A0A3G2JVM7	42519394	OppA	ABC transporter system
A0A4Y9IGT3	42519448	OppA	ABC transporter system
A0A7D9N5R4	42519448	OppA	ABC transporter system
F4AG66	42519448	OppA	ABC transporter system
Q74HU5	42519699	OppA	ABC transporter system
Q74IJ4	42519448	OppA	ABC transporter system
Q74IP6	42519395	OppA	ABC transporter system
Q74IP7	42519394	OppA	ABC transporter system
A0A137PMA8	42519469	PepM	Aminopeptidase
A0A355X176	42518488	PepC	Aminopeptidase
A0A4Y9ICH0	42518641	PepC	Aminopeptidase
A0A4Y9IDT6	42519847	Pcp	Aminopeptidase
Q74HE9	42519847	Pcp	Aminopeptidase
Q74IH3	42519469	PepM	Aminopeptidase
Q74II4	42519458	PepN	Aminopeptidase
Q74KM5	42518649	PepA	Aminopeptidase
Q74KN3	42518641	PepC	Aminopeptidase
Q74KN6	42518638	PepC	Aminopeptidase
Q74L31	42518488	PepC	Aminopeptidase
P60810	42519890	PrtM	cell-wall bound proteinase
Q74HA7	42519889	PrtP	cell-wall bound proteinase
Q74KK7	42518667	DtpT	di/tripeptides uptaken
A0A137PNU7	42518589	PepV	dipeptidase
A0A1B3PW14	42518668	PepD	dipeptidase
A0A244CDU7	42519390	PepD	dipeptidase
F4AFA0	42518589	PepV	dipeptidase
Q74HL4	42519780	PepD	dipeptidase
Q74HX2	42519672	PepD	dipeptidase

Q74IQ1	42519390	PepD	dipeptidase
Q74KK6	42518668	PepD	dipeptidase
Q74KN4	42518640	PepD	dipeptidase
Q74KT4	42518589	PepV	dipeptidase
Q74LC6	42518345	PepD	dipeptidase
A0A137PNQ2	42518264	PepE/PepG	endopeptidase
A0A4Y9IF91	42519446	PepE/PepG	endopeptidase
Q74HQ0	42519744	PepF	endopeptidase
Q74IJ6	42519446	PepE/PepG	endopeptidase
Q74J14	42519221	PepO	endopeptidase
Q74LK7	42518264	PepE/PepG	endopeptidase
Q74LK9	42518262	PepE/PepG	endopeptidase
Q74LN6	42518233	PepO	endopeptidase
Q74M04	42518115	PepO	endopeptidase
A0A1B3PWC6	42519399	OppD	oligopeptides uptake
A0A1B3PWD2	42519398	OppF	oligopeptides uptake
A0A267M6Z5	42519396	OppC	oligopeptides uptake
A0A267M7N0	42519397	OppB	oligopeptides uptake
F4AGJ2	42519399	OppD	oligopeptides uptake
F4AGJ3	42519398	OppF	oligopeptides uptake
Q74IP2	42519399	OppD	oligopeptides uptake
Q74IP3	42519398	OppF	oligopeptides uptake
Q74IP4	42519397	OppB	oligopeptides uptake
Q74IP5	42519396	OppC	oligopeptides uptake
A0A244CDX9	42519426	PepP	Proline peptidase
A0A244CJQ4	42518140	PepR	Proline peptidase
A0A3G2JVS5	42519459	PepX	Proline peptidase
A0A4Y9ICR3	42518586	PepQ	Proline peptidase
F4AG55	42519459	PepX	Proline peptidase
Q74II3	42519459	PepX	Proline peptidase
Q74IL6	42519426	PepP	Proline peptidase

Q74KT7	42518586	PepQ	Proline peptidase
Q74LX9	42518140	PepR	Proline peptidase
A0A137PPM8	42519228	PepT	tripeptidase
A0A6P1Y878	42519680	PepT	tripeptidase
A0A7D9N7R4	42519680	PepT	tripeptidase
Q74HW4	42519680	PepT	tripeptidase
Q74J07	42519228	PepT	tripeptidase

Table S8. Evaluation of Peptide Sequences from Pea Protein Hydrolysates for Sensory Attributes Using iUmami and iBitter Sequence-Composition Models (SCM).[7,8] This table includes peptide length, fraction assignment, umami scores, predicted umami attributes, bitterness scores, and predicted bitterness attributes for sequences identified exclusively in the 48-hour fermented taste-active SPE fractions F1 and F2, except for the sequence ANAQPLQRE, which was unique to the SPE unfermented fraction U2

Sequence	Lenght	fraction	iUmami SCM score	iUmami SCM predicted attribute	iBitterSCM score	iBitter SCM
GQIEEL	6	F1	611.2	Umami	304.4	non-Bitter
GSAQEVD	7	F1	630.17	Umami	323.67	non-Bitter
EVDRLLKN	8	F2	593.43	Umami	306.86	non-Bitter
GQIEELSKN	9	F2	644.88	Umami	313.38	non-Bitter
GSSHEVD	7	F1	624.33	Umami	326.83	non-Bitter
ELTPE	5	F1	637.5	Umami	352.25	Bitter
AGEEDNVIS	9	F1	660.75	Umami	413.25	Bitter
EENVIVKV	8	F2	631.57	Umami	363.14	Bitter
REQIEEL	7	F1	677.83	Umami	348.67	Bitter
SREQIEEL	8	F1	673.86	Umami	346.71	Bitter
DKEEEQEEETSKQVQ	15	F1	716.21	Umami	342.64	Bitter
ANAQPLQRE	9	U2	585.5	non-Umami	341.88	Bitter

Table S9. BLAST scores, raw data. The provided table outlines the results obtained from the results obteained from the process where protein sequences were search through BLAST (Basic Local Alignment Search Tool) to compare against genomes of *L. johnsonii* strains, focusing on identifying the presence of specific genes within these strains. The criteria for selecting significant BLAST hits included e-value threshold of 0.001 and a minimum coverage of 80% relative to the query sequence. These data points, were then used to construct heatmaps, providing a visual representation of the genetic similarity and diversity among the *L. johnsonii* strains tested.

Gene	NCC1584	NCC1657	NCC1680	NCC2680	NCC3033	NCC533
P60810	44.238	100	44.61	100	96.98	100
Q74HE9	99.07	100	97.674	100	95.814	100
A0A137PMA8	99.635	100	99.27	100	98.175	100
A0A137PNQ2	99.541	100	99.312	100	97.936	100
A0A137PNU7	99.785	100	99.14	100	98.065	100
A0A137PPM8	99.759	100	99.036	100	98.072	100
A0A1B3PW14	100	100	59.914	100	92.949	100
A0A1B3PWC6	99.702	99.702	99.107	99.702	98.512	99.702
A0A1B3PWD2	100	100	98.137	100	97.516	100
A0A244CDU7	100	100	98.523	100	96.835	100
A0A244CDX9	100	100	99.187	100	93.767	100
A0A244CJQ4	100	100	99.342	100	96.711	100
A0A267M6Z5	99.671	100	97.368	100	93.75	100
A0A267M7N0	100	100	97.377	100	97.705	100
A0A355X176	100	100	98.886	100	100	100
A0A3G2JVM7	99.825	100	98.953	100	98.255	100
A0A3G2JVS5	98.741	100	98.741	100	96.096	100
A0A4Y9ICH0	97.279	100	77.626	100	91.837	100
A0A4Y9ICR3	98.641	100	98.913	100	95.924	100
A0A4Y9IDT6	99.07	100	97.674	100	95.814	100
A0A4Y9IF91	99.543	100	97.717	100	94.749	100
A0A4Y9IGT3	59.829	100	97.089	100	89.555	100
A0A6P1Y878	97.877	100	96.226	100	86.792	100
A0A7D9N5R4	59.829	100	97.089	100	89.555	100
A0A7D9N7R4	97.877	100	96.226	100	86.792	100
F4AFA0	99.785	100	99.14	100	98.065	100
F4AG55	98.741	100	98.741	100	96.096	100
F4AG66	59.829	100	97.089	100	89.555	100
F4AGJ2	99.702	99.702	99.107	99.702	98.512	99.702
F4AGJ3	100	100	98.137	100	97.516	100
Q74HA7	0	100	0	100	82.944	100
Q74HL4	99.149	100	96.383	100	91.277	100
Q74HQ0	99.169	100	97.674	100	89.149	100
Q74HU5	98.459	100	96.364	100	94.027	100
Q74HW4	97.877	100	96.226	100	86.792	100
Q74HX2	98.742	100	98.113	100	95.388	100
Q74IH3	99.635	100	99.27	100	98.175	100
Q74II3	98.741	100	98.741	100	96.096	100

Q74II4	99.052	100	98.815	100	94.431	100
Q74IJ4	59.829	100	97.089	100	89.555	100
Q74IJ6	99.543	100	97.717	100	94.749	100
Q74IL6	100	100	99.187	100	93.767	100
Q74IP2	99.702	99.702	99.107	99.702	98.512	99.702
Q74IP3	100	100	98.137	100	97.516	100
Q74IP4	100	100	97.377	100	97.705	100
Q74IP5	99.671	100	97.368	100	93.75	100
Q74IP6	95.034	100	98.288	100	71.453	100
Q74IP7	99.825	100	98.953	100	98.255	100
Q74IQ1	100	100	98.523	100	96.835	100
Q74J07	99.759	100	99.036	100	98.072	100
Q74J14	27.579	99.443	23.251	99.443	98.145	99.443
Q74KK6	100	100	59.914	100	92.949	100
Q74KK7	99.195	100	98.793	100	97.183	100
Q74KM5	100	100	99.167	100	0	100
Q74KN3	97.279	100	77.626	100	91.837	100
Q74KN4	97.046	100	43.312	100	94.726	100
Q74KN6	99.546	100	88.209	100	86.652	100
Q74KT4	99.785	100	99.14	100	98.065	100
Q74KT7	98.641	100	98.913	100	95.924	100
Q74L31	100	100	98.886	100	100	100
Q74LC6	100	100	98.938	100	97.028	100
Q74LK7	99.541	100	99.312	100	97.936	100
Q74LK9	97.025	100	98.398	100	94.279	100
Q74LN6	99.23	100	99.23	100	95.532	100
Q74LX9	100	100	99.342	100	96.711	100
Q74M04	51.389	100	98.113	100	51.562	100

REFERENCES

(1) MacLean, B.; Tomazela, D. M.; Shulman, N.; Chambers, M.; Finney, G. L.; Frewen, B.; Kern, R.; Tabb, D. L.; Liebler, D. C.; MacCoss, M. J. Skyline: An Open Source Document Editor for Creating and Analyzing Targeted Proteomics Experiments. *Bioinformatics* **2010**, *26* (7), 966–968. https://doi.org/10.1093/bioinformatics/btq054.

(2) Toelstede, S.; Hofmann, T. Quantitative Studies and Taste Re-Engineering Experiments toward the Decoding of the Nonvolatile Sensometabolome of Gouda Cheese. *J Agric Food Chem* **2008**, *56* (13), 5299–5307.

(3) Rotzoll, N.; Dunkel, A.; Hofmann, T. Activity-Guided Identification of (S)-Malic Acid 1-O-D-Glucopyranoside (Morelid) and γ-Aminobutyric Acid as Contributors to Umami Taste and Mouth-Drying Oral Sensation of Morel Mushrooms (Morchella Deliciosa Fr.). *J Agric Food Chem* **2005**, *53* (10), 4149–4156. https://doi.org/10.1021/jf050056i.

(4) Schindler, A.; Dunkel, A.; Stähler, F.; Backes, M.; Ley, J.; Meyerhof, W.; Hofmann, T. Discovery of Salt Taste Enhancing Arginyl Dipeptides in Protein Digests and Fermented Fish Sauces by Means of a Sensomics Approach. *J Agric Food Chem* **2011**, *59* (23), 12578–12588. https://doi.org/10.1021/jf2041593.

(5) Dunkel, A.; Hofmann, T. Sensory-Directed Identification of β-Alanyl Dipeptides as Contributors to the Thick-Sour and White-Meaty Orosensation Induced by Chicken Broth. *J Agric Food Chem* **2009**, *57* (21), 9867–9877. https://doi.org/10.1021/jf900948r.

(6) Salger, M.; Stark, T. D.; Hofmann, T. Taste Modulating Peptides from Overfermented Cocoa Beans. *J Agric Food Chem* **2019**, *67* (15), 4311–4320. https://doi.org/10.1021/acs.jafc.9b00905.

(7) Charoenkwan, P.; Yana, J.; Schaduangrat, N.; Nantasenamat, C.; Hasan, M. M.; Shoombuatong, W. IBitter-SCM: Identification and Characterization of Bitter Peptides Using a Scoring Card Method with Propensity Scores of Dipeptides. *Genomics* **2020**, *112* (4), 2813–2822. https://doi.org/10.1016/j.ygeno.2020.03.019.

(8) Charoenkwan, P.; Yana, J.; Nantasenamat, C.; Hasan, M. M.; Shoombuatong, W. IUmami-SCM: A Novel Sequence-Based Predictor for Prediction and Analysis of Umami Peptides Using a Scoring Card Method with Propensity Scores of Dipeptides. *J Chem Inf Model* **2020**, *60* (12), 6666–6678. https://doi.org/10.1021/acs.jcim.0c00707.
